# Part 2: Stabilization/Containment of Radiological Particle Contamination to Enhance First Responder, Early Phase Worker, and Public Safety

**DOI:** 10.3390/app12083861

**Published:** 2022-04-11

**Authors:** Matthew Magnuson, Terry Stilman, Shannon Serre, John Archer, Ryan James, Xiaoyan Xia, Mitchell Lawrence, Erin Tamargo, Hadas Raveh-Amit, Avi Sharon

**Affiliations:** 1 EPA Office of Research and Development, Homeland Security Materials and Management Division, 26 W Martin Luther King Dr., Cincinnati, OH 45268, USA; 2 EPA Region 4, 61 Forsyth St., SW, Atlanta, GA 30303, USA; 3 EPA Office of Land and Emergency Management, Consequence Management Advisory Division, WJC-N, Washington, DC 20002, USA; 4 Battelle Memorial Institute, 505 King Ave., Columbus, OH 43201, USA; 5 Irregular Warfare Technical Support Directorate, Alexandria, VA 22350, USA; 6 Department of Chemistry, Nuclear Research Centre Negev, P.O. Box 9001, Beer Sheva 8419000, Israel; 7 Environmental Research Unit, Nuclear Research Centre Negev, P.O. Box 9001, Beer Sheva 8419000, Israel

**Keywords:** stabilization, containment, radiological contamination, cesium, strontium

## Abstract

The application of stabilization technologies to a radiologically contaminated surface has the potential for reducing the spread of contamination and, as a result, decreasing worker exposure to radiation. Three stabilization technologies, calcium chloride (CaCl_2_), flame retardant Phos-Chek^®^ MVP-Fx, and Soil_2_O™ were investigated to evaluate their ability to reduce the resuspension and tracking of radiological contamination during response activities such as vehicle and foot traffic. Concrete pavers, asphalt pavers, and sandy soil walking paths were used as test surfaces, along with simulated fallout material (SFM) tagged with radiostrontium (Sr-85) applied as the contaminant. Radiological activities were measured using gamma spectrometry before and after simulated vehicle operation and foot traffic experiments, conducted with each stabilization technology and without application as a nonstabilized control. These measurements were acquired separately for each combination of surface and vehicle/foot traffic experiment. The resulting data describes the extent of SFM removed from each surface onto the tires or boots, the extent of SFM transferred to adjacent surfaces, and the residual SFM remaining on the tires or boots after each experiment. The type of surface and response worker actions influenced the stabilization results. For instance, when walked over, less than 2% of particles were removed from nonstabilized concrete, 4% from asphalt, and 40% of the particles were removed from the sand surface. By contrast, for vehicle experiments, ~40% of particles were again removed from the sand, but 7% and 15% from concrete and asphalt, respectively. In most cases, the stabilization technologies did provide improved stabilization. The improvement was related to the type of surface, worker actions, and stabilizer; a statistical analysis of these variables is presented. Overall, the results suggest an ability to utilize these technologies during the planning and implementation of response activities involving foot and vehicle traffic. In addition, resuspension of aerosolizable range SFM was monitored during walking path foot traffic experiments, and all stabilizing agents decreased the measured radioactivity, with the Soil_2_O™ decrease being 3 fold, whereas the CaCl_2_ and Phos-Chek MVP-Fx surfaces generated no detectable radioactivity. Overall, these results suggest that the stabilization technologies decrease the availability of particles respirable by response workers under these conditions.

## 1. Introduction

Discharge of a radiological dispersal device (RDD) or an accidental radiological release may cause contamination over a wide area. During response and recovery, emergency responders, recovery workers, and the general public may be exposed to radioactivity due to direct contact and inhalation. Direct contact results from “hands-on” activities with contaminated material or exposure to unshielded radiation sources. Inhalation exposure could originate from dust generated by the release incident or during demolition or decontamination work [1], as well as from the resuspension of such dust during work activities. The risk of tracking contamination via vehicle or foot traffic is of significant concern. Tracking can lead to contaminant spread which enlarges the contaminated area, potentially increasing the time and expense of decontamination efforts.

Control of the contamination source area to prevent resuspension and tracking could both reduce radioactivity exposure and reduce the spread of contamination. Hence, there is a need for technologies and methodologies to limit exposure, reduce resuspension, and limit the tracking of radioactive materials. Such technologies have been widely investigated for nonradioactive materials due to the importance of controlling urban particulate matter and its associated human health effects. These technologies broadly fall into two categories: physical removal and stabilization [1–4]. Physical removal approaches include sweeping, washing, wiping, and vacuuming. Stabilization technologies prevent particles from spreading by resuspension or tracking, and such technologies are routinely used for dust control in industries such as road construction, and at mining sites.

Technologies for preventing secondary transport of soluble and particulate radiological contamination from roadways, roadside vegetation, and adjacent soils have recently been reviewed by Saito [5]. Although the focus of the review was preventing resuspension associated with decontamination activities, not necessarily resuspension arising directly from vehicle and foot traffic, it suggests that widely available and easily applicable stabilization technologies have the potential to minimize worker exposure by reducing the spread of contamination. These technologies may also minimize the resuspension of radioactive particles from surfaces, thus reducing the time and resources needed for additional decontamination operations [5]. Technologies for minimizing resuspension are an active research area because of keen interest in controlling dust and other particulate matter from anthropogenic sources due to its potential impact on human health [3,6–12].

In a previous study, the U.S. Environmental Protection Agency (EPA) assembled stakeholders with expertise in radiological stabilization and performed a downselection exercise to prioritize experimental testing of stabilization technologies (e.g., water, fire retardants, gels, foams, and clays) based on literature searches and the personal experience of the stakeholders [13]. Twenty-four technologies were identified, and, based on stakeholder ranking, a fire retardant (Phos-Chek MVP-F) and two dust suppression technologies (Soil_2_O and CaCl_2_) were downselected to evaluate the efficacy of particle stabilization during vehicle and foot traffic. This evaluation determined that cesium (Cs)-137 quantitatively bound to both Phos-Chek MVP-F and Soil_2_O, while CaCl_2_ application increased sorption of aqueous Cs-137 onto the surface matrix, Arizona road dust. During driving activities, the transfer of particles from treated surfaces was least for Phos-Chek MVP-F fire retardant, followed by Soil_2_O. The transfer of particles was greatest for surfaces treated with CaCl_2_. An evaluation of the impacts on decontamination processes, waste generation, and the environment following stabilization suggested that Phos-Chek MVP-F, CaCl_2_, and Soil_2_O demonstrated the feasibility of using these materials, traditionally used for other purposes, for radiological stabilization [13]. A second EPA project evaluated the same technologies qualitatively, and the results indicated that the Phos-Chek MVP-F was more effective at particle stabilization than Soil_2_O and CaCl_2_ [14].

A recent collaborative effort between the EPA and the Nuclear Research Centre Negev (NRCN) studied wind-induced resuspension. It highlighted the need to validate stabilization technologies for the physical stresses from specific resuspension processes other than wind because these physical stresses lead to the disintegration of stabilized particles and, ultimately, resuspension [15]. This paper builds on that collaboration and includes the Irregular Warfare Technical Support Directorate [16]. This paper uses pilot-scale experiments with radiolabeled simulated fallout material (SFM) to investigate stabilization efficacy against specific stresses related to vehicle and foot traffic on surfaces of interest for resuspension and tracking, especially during urban response and recovery. The tests are designed to describe the extent of particle removal from each surface onto the tires of vehicles or the boots of responders, the extent of SFM transferred to adjacent surfaces, and the residual SFM remaining on the tires or boots after each experiment. Other tests investigate the resuspension of aerosolizable range particles potentially subject to inhalation during foot traffic.

## 2. Materials and Methods

### 2.1. Description of Stabilization Materials

CaCl_2_ (CESCO Solutions Inc., Bellingham, WA, USA), Phos-Chek MVP-Fx (Perimeter Solutions, Rancho Cucamonga, CA, USA), and Soil_2_O (Geltech Solution, Jupiter, FL, USA) were investigated in this study. The Supplemental Information includes information sheets that summarize operationally important aspects of these materials. Briefly, CaCl_2_ (Table S1) is a hygroscopic material attracting moisture from the air that works to increase the moisture level on surface particles, decreasing particle resuspension and transfer. CaCl_2_ is applied as a solution, which, upon drying, creates a weak cementation effect by binding fine particulates together. This moisture binds fine aggregate particles to decrease particle resuspension and transfer. CaCl_2_ is extensively used for dust control during construction projects and on unpaved roads, as it is easy to use and widely available. Phos-Chek MVP-Fx (Table S2) is a gum-thickened, medium viscosity fire retardant that provides accurate aerial drops for wildland fire control in forest, bush, or grassland. With added colorant, Phos-Chek MVP-Fx is a high visibility powder concentrate that can readily be mixed with water. Soil_2_O (Table S3) is a dust control agent that can effectively suppress particulate matter from entering the air. Soil_2_O is a copolymer intended to maintain the moisture level in the soil by preventing evaporation, thus decreasing the propensity of particles to be available for dust formation.

### 2.2. Experimental Procedure

#### 2.2.1. Preparation of Simulated Fallout Material (SFM) Containing Radiostrontim (Sr-85)

Arizona test dust with a particle size ranging from a few micrometers (μm) to a few hundred μm (Powder Technology Inc., Arden Hills, MN, USA) was selected to be used in the evaluation testing since the particles in selected particle size ranges were susceptible to resuspension and tracking. To prepare the mixture of particles, equal amounts of ultrafine test dust (ISO 12103–1, A1) and coarse test dust (ISO 12103–1, A4) were mixed. To make radioactive Sr-85 contaminated particles, an aqueous solution that contained 500 microcuries (μCi) Sr-85 (strontium chloride in 0.5 M HCl, Eckert & Ziegler Isotope Products, Valencia, CA) was added to ~250 g (g) of substrate particles using a sprayer (BS-3 3 oz Locking Personal Sprayer, Sprayco, Liwnia, MI, USA). The particles were wellmixed by manually shaking the container side to side for approximately 10 s and then manually rotating the container for approximately 30 s. This process was repeated until the required volume of aqueous Sr-85 was added to the particles. After being allowed to dry overnight in a well-ventilated exhausted box in a fume hood (T: 22.0 ± 0.7 °C; RH: 24.9 ± 0.5%), the spiked substrate particles were mixed again for approximately 10 min to ensure a homogenous mixture. Then, the dry particles were transferred to saltshakers (Tablecraft Products Company, 3-oz glass with stainless steel top, UNSPSC# 52152013), used for application. For simulated vehicle experiments and walking experiments on concrete and asphalt surfaces, ~3 g of particles were transferred from each saltshaker to contaminate one testing surface. For walking experiments on the soil walking path surface, ~40 g of particles were transferred from each saltshaker for one experiment.

To characterize the particle size of the contaminated particles and confirm the lack of particle clumping during contamination, an identical particle mixture was prepared, and one aliquot was wetted with non-radiological aqueous solvent and then dried using a procedure similar to the procedure for the preparation of radiological particles. The particle size was determined for both aliquots using a standard test method for particle-size analysis of soils, ASTM D422–63. The analytical results of the particle sizes are listed in Table 1, and the wetted and nonwetted samples produced similar results.

#### 2.2.2. Preparation of Surfaces for Testing

The contaminated fallout material was applied to three surfaces for the simulated vehicle-rolling and foot-traffic experiments. These surfaces included asphalt pavers (6″ × 12″ × 1.75″, Asphalt Products, Hanover, PA, USA), concrete pavers (12″ × 12″ × 1.75″, Pewter Square Concrete Step Stone, The Home Depot, Atlanta, GA, USA), and sand (Garick premium play sand, Garick LLC, Cleveland, OH, USA) loosely placed on vinyl fabric (80 Vinyl Military Spec Fabric, Herculite Products Inc., Emigsville, PA, USA) covering the floor of a tent. All the pavers were purchased in bulk and used in as-received condition. The soil bags were opened in the laboratory a few days before use; thus, the soil was equilibrated to laboratory conditions (T: 21.9 ± 0.7 °C and RH: 54 ± 7%).

The Garick sand utilized was selected as follows: Two (2) Israeli sandy soil samples, including “Ze’elim” and “Rotem”, were received and characterized. Two (2) local soil samples, including Ohio mulch (Ohio Mulch, Columbus, OH, USA) and the Garick sand with similar particle sizes, were characterized and compared at the same time. The test results are listed in Table 2.

Garick sand was selected for the study because of its particle sizes in the sand fraction, similar to Rotem particle sizes. While this sand is technically a “soil”, the material will be referred to throughout this document as a “sand surface” because the surface is primarily sand and also because one of the stabilization products is called “Soil_2_O” which tends to cause some linguistic confusion.

#### 2.2.3. Preparation of Stabilization Technologies

Based on manufacturers’ instructions and previous studies [13,14], aqueous solutions were prepared for each stabilization technology for the dry runs, and the concentrations were finalized for actual testing. The preparation procedure for each stabilization technology is summarized below. The deionized (DI) water was from a Barnstead DI water system (D12681, Barnstead International, Dubuque, IA, USA).

CaCl_2_ solution was prepared by weighing 100 g of CaCl_2_ pellets and transferring them to a 2 L volumetric flask. The flask was partially filled with DI water and mixed until solids were completely dissolved. Then, the flask was filled to the volume of 1600 mL with DI water and mixed again until the solution was homogenous. Soil_2_O (5 g) was weighed and transferred to a 2 L volumetric flask. The flask was partially filled with DI water and mixed until the powder was dissolved. Then, the flask was filled to a volume of 1600 mL with DI water and mixed again until the solution was homogenous. Phos-Chek MVP-Fx (110 g) was weighed and transferred to a 1 L volumetric flask. The flask was filled partially with DI water and mixed until solids were completely wet, and then the flask was filled to the 1 L volume with DI water and mixed well.

#### 2.2.4. Radiological Contamination and Stabilization of Surfaces

**Figure 1.**
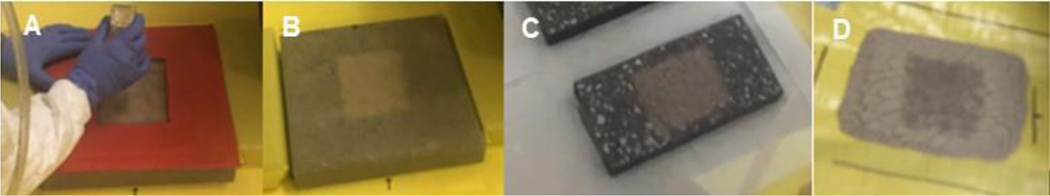
Application of simulated fallout material (SFM) to tested surfaces. (**A**) Use of stencil to define target area; (**B**) Concrete; (**C**) Asphalt; (**D**) Sand.

In an actual fallout event, the material loading level would vary greatly depending on the height of a possible explosion, ground characteristics below a possible explosion, distance from the release point, meteorological conditions, and ventilation of residences or offices. Previous fallout decontamination research [17,18], mostly outdoor, has used surface densities of approximately 20 mg/cm^2^, so a similar density was used as the surface loading. To apply contaminated particles to the testing surface, approximately 3 g of particles were measured and transferred into a saltshaker and rotated to mix well. One shaker was emptied onto each target area, where foot or tire contact was made of the simulated traffic surface (12.5 cm by 15 cm) corresponding to 16 mg/cm^2^ and 5 to 6 μCi of Sr-85. For walking experiments on the sand walking path surface, 40 g of contaminated particles was applied to each target area (45 cm by 45 cm), corresponding to 20 mg/cm^2^ and 80 μCi of Sr-85. The contaminated concrete, asphalt, and sand walking path surfaces are shown in Figure 1.

#### 2.2.5. Measurement of Sr-85 Activity

**Figure 2.**
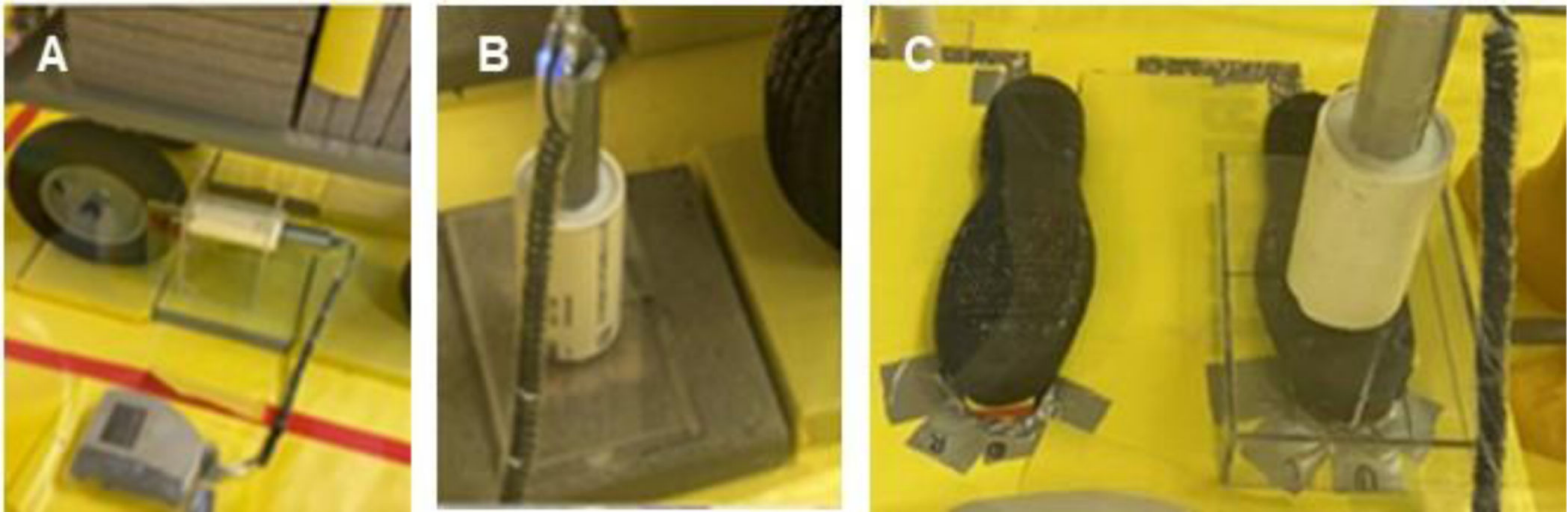
Activity Measurement Using Frames Customized to (**A**) Tires; (**B**) Surfaces; and (**C**) Boots.

The measurement of gamma radiation of Sr-85 from the testing surfaces was performed using a sodium iodide (NaI) spectrometer (Canberra InSpector 1000, Canberra Industries, Inc., Meriden, CT, USA) with different customized stand frames (Figure 2) made of Plexiglass^®^ to ensure a constant distance (1.27 cm) between the detector and the measured surfaces. After applying Sr-85 contaminated particles to the testing surface or following the application of stabilization technologies on the contaminated surfaces, the center of the contaminated area was measured for the activity of Sr-85. To track the transfer of Sr-85 as the result of the movement of simulated vehicles and walking, the contaminated/stabilized surfaces, transferred surfaces that were previously uncontaminated surfaces, and the tires/boots were measured for the activity of Sr-85 after rolling simulated vehicle/walking across the surfaces. The instrument performance testing of Canberra InSpector 1000 was conducted daily and met the acceptance criteria. Specifically, the instrument’s accuracy was monitored through daily performance checks, which included analysis of a sealed sample of 1 μCi Sr-85. The sealed sample was analyzed in the same geometry with respect to the probe with a 100-s acquisition time. The instrument performance was considered consistent if the relative percent difference (RPD) of the measured activity and the theoretical value of Sr-85 prepared due to decay were within 10% throughout the testing. Based on the half-life of Sr-85 (64.8 days), the theoretical values were calculated on each experiment day. Across the two months of testing, the RPD ranged from −2.4% to 2.5%, indicating the consistency of instrument performance.

#### 2.2.6. Evaluation of Stabilization Technologies

**Figure 3.**
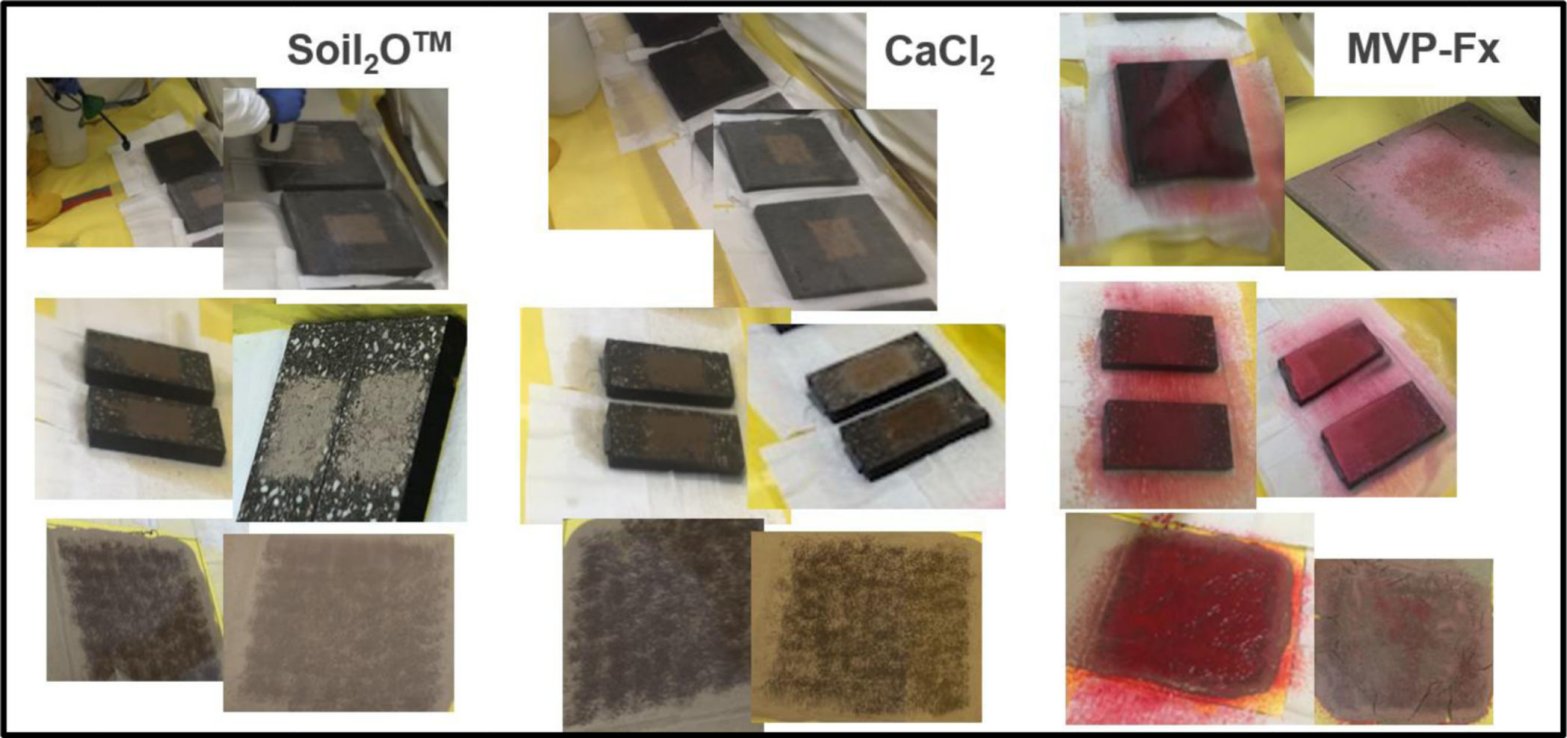
Surfaces Treated with Stabilization Technologies (Left: Wet; Right: Dry).

The stabilization technologies were applied to the surfaces studied as described below, and the exact application varied with the type of experiment and the specific surfaces involved. As an example, Figure 3 shows the stabilization technologies for treated surfaces before and after drying. Figure 3 is intended to highlight the appearance of the technologies, whereas later figures illustrate the steps of evaluating the technologies.

**Figure 4.**
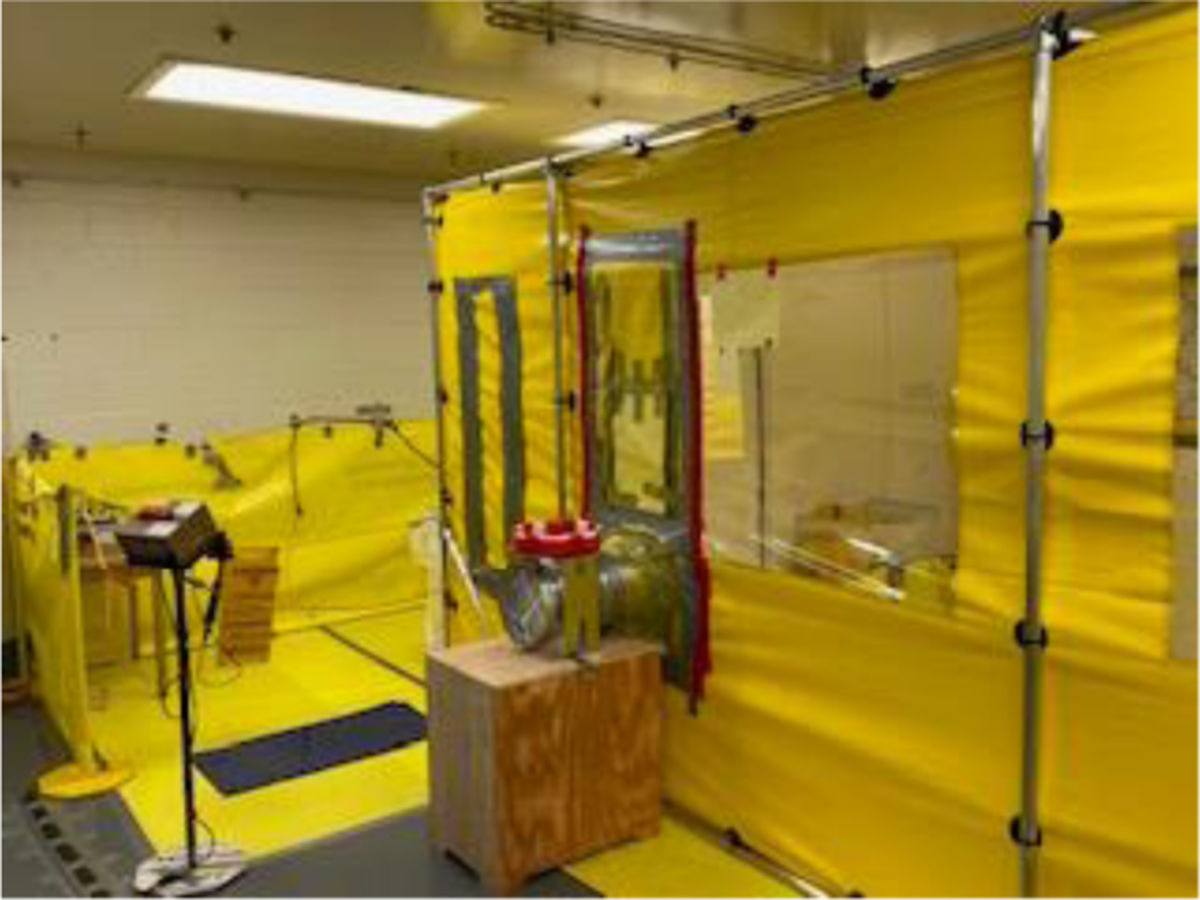
Radiological Containment Tent.

Simulated vehicle experiments and walking experiments were conducted on the three surfaces treated with three stabilization technologies on each surface. Quality control (QC) samples included background and nonstabilized controls (NSCs). All the radiological stabilization technology testing experiments were performed in a containment tent (Dual Chamber Tent, LANCS Industries, Kirkland, WA, (15 ft long × 8 ft wide × 7 ft high) as shown in Figure 4, which was located in a laboratory. The temperature and relative humidity (RH) were continuously monitored in the tent, and the data were stored in a HOBO data logger. Throughout stabilization technology evaluation experiments, the average temperature and RH in the tent were 21.9 ± 0.7 °C and 53.5% ± 6.6%, respectively.

**Figure 5.**
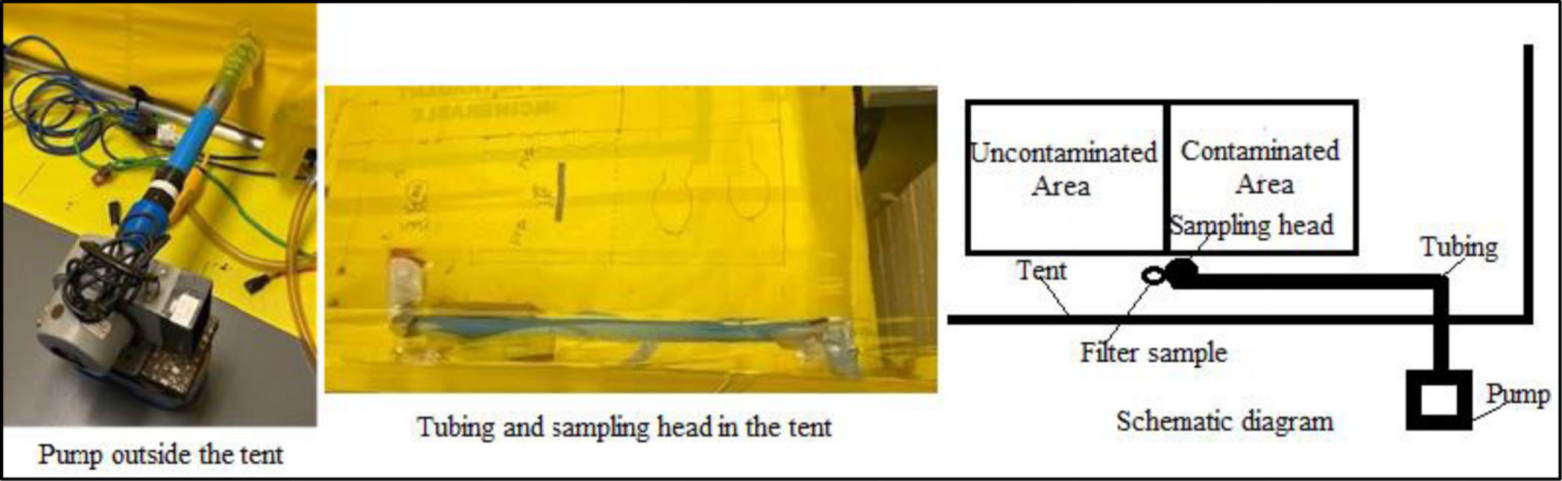
Schematic Diagram of High-Volume Sampler and Filter Sample Setup (**left**); with pictures of the pump (**right**) and tubing and sampling head (**middle**) to show a relationship to the containment tent.

The radiological testing technicians wore personal protective equipment (PPE) that included protective coveralls, gloves, shoe covers, boots, powered air-purifying respirators, and a personal air sampler (AirChek 52, SKC Inc., 863 Valley View Road, Eighty-Four, PA, USA). The AirChek 52 personal air sampler collected particulates on a glass fiber filter during each experiment. The filters were analyzed for Sr-85 activity to monitor for possible inhalational exposure to the radiological testing technician (all results were below background levels). The tent was connected to a high-efficiency particle air filtration system, and two air sampling systems (LV-10, F&J Specialty Products Inc., Ocala, FL, USA) were operated at 3 cubic feet per minute (cfm) to collect air samples on 47 mL round quartz fiber filters within the radiological containment tent during experiments. As shown in Figure 5, an additional air sampling system (HV-1, F&J Specialty Products, Inc., Ocala, FL, USA) operating at approximately 5 cfm was used during walking experiments on the sand walking path surfaces at low height (~30 cm above the contaminated sand surface) to collect air samples and characterize particle resuspension of aerosolizable range particles. The sampling system utilized 47 mm FP-XM glass fiber filters (F&J Specialty Products, Inc., Ocala, FL, USA).

#### 2.2.7. Simulated Vehicle Experiments

**Figure 6.**
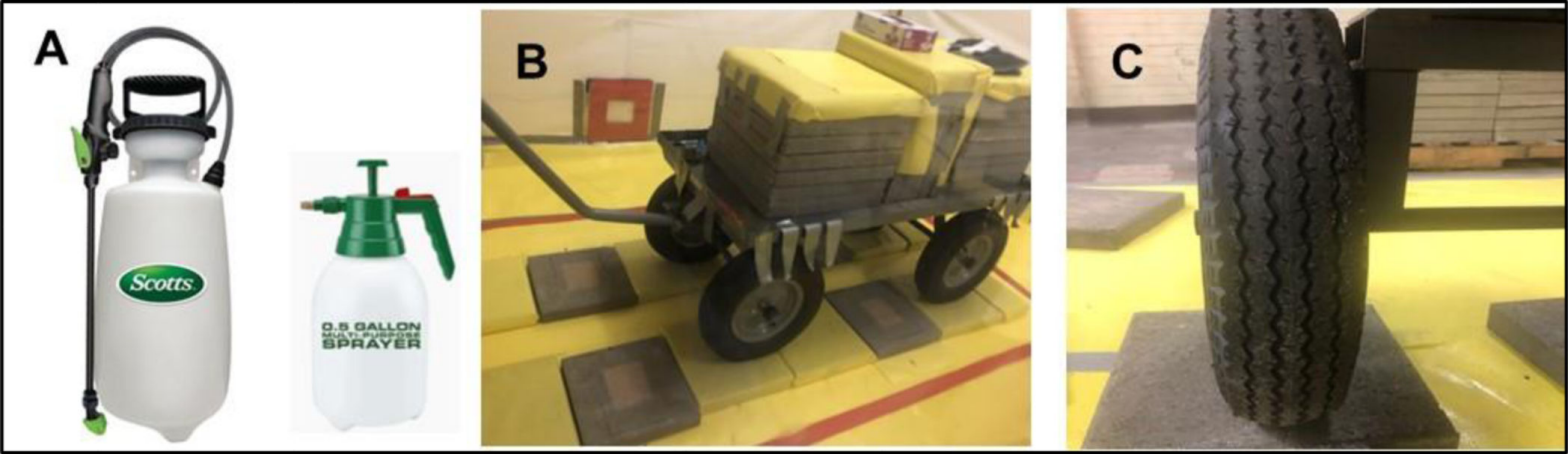
Sprayers Used in Study (**A**), Wagon Used to Simulate Applicable Vehicle (**B**), and Simulated Vehicle Tire (**C**).

For simulated vehicle experiments, approximately 3 g of Sr-85 contaminated particles was applied to each testing surface with a surface area of 12.5 cm × 15 cm (using a template to maintain repeatable particle coverage). Four (4) contaminated surfaces without the application of any stabilization technologies were prepared for each testing surface as NSCs. Uncontaminated surfaces served as blanks for background measurements. After the surface was contaminated with Sr-85, a stabilization technology solution was sprayed onto the contaminated surfaces (Figure 6A) and allowed to dry in the tent (drying time ranged from overnight to 3 days, as the surfaces did not all seem to dry completely overnight). CaCl_2_ and Soil_2_O solutions were sprayed using a two-gallon multipurpose pump-up Scotts sprayer with the cone spray setting (Item 190498, https://www.scotts.com, accessed on 9 April 2022). Phos-Chek MVP-Fx solution was sprayed using a 0.5-gallon multipurpose handheld pump-up sprayer (Item 56167, http://www.harborfreight.com, accessed on 9 April 2022) because the other sprayer would quickly clog while using Phos-Chek MVP-Fx. Four (4) contaminated surfaces with each stabilization technology were prepared for each testing surface material. A small wagon (Model # H-2547; deck size: 24″ × 48″, load capacity: 3000 lb; wheel type: 16″ pneumatic; https://www.uline.com/, accessed on 9 April 2022) fitted with pneumatic tires with tread comparable to applicable vehicle tires (Figure 6B) was loaded with 800 lb of concrete pavers that generated a contact pressure similar to applicable vehicles with downforce of 2000 lb. The evaluation testing for one testing surface material was set up under four tires simultaneously (Figure 6C). The wagon was manually pulled to move across contaminated/stabilized surfaces, as shown in Video S1 in Supplemental Information. The radiological activities of the contaminated/stabilized surfaces were measured before and after simulated vehicle rolling.

The contaminated surfaces were replaced with uncontaminated surfaces, and the wagon was pushed backward across the uncontaminated transfer surfaces. The radiological activities of the previously uncontaminated and post-traffic contaminant transfer surfaces were measured to evaluate the magnitude of Sr-85 transfer. The radiological activities of Sr-85 from the tires having contacted the center of the contaminated/stabilized surfaces were measured to quantify the residual of Sr-85 contaminated particles on the tire. All measurements were done using an NaI spectrometer with different customized stand frames made of Plexiglass^®^ to ensure a constant distance (1.27 cm) between the detector and the measured surfaces. For testing experiments on sand surfaces, both contaminated/stabilized and transferred surfaces were prepared by filling a 25 cm × 25 cm template on a piece of Herculite^®^ plastic sheeting (Emigsville, PA, USA) with equal amounts of premeasured sand. Then, the sand surfaces were contaminated/stabilized the same way as the concrete and asphalt pavers. The same rolling procedures were applied to sand surfaces, and the corresponding measurements were performed.

#### 2.2.8. Straight-Line Walking Experiments

**Figure 7.**
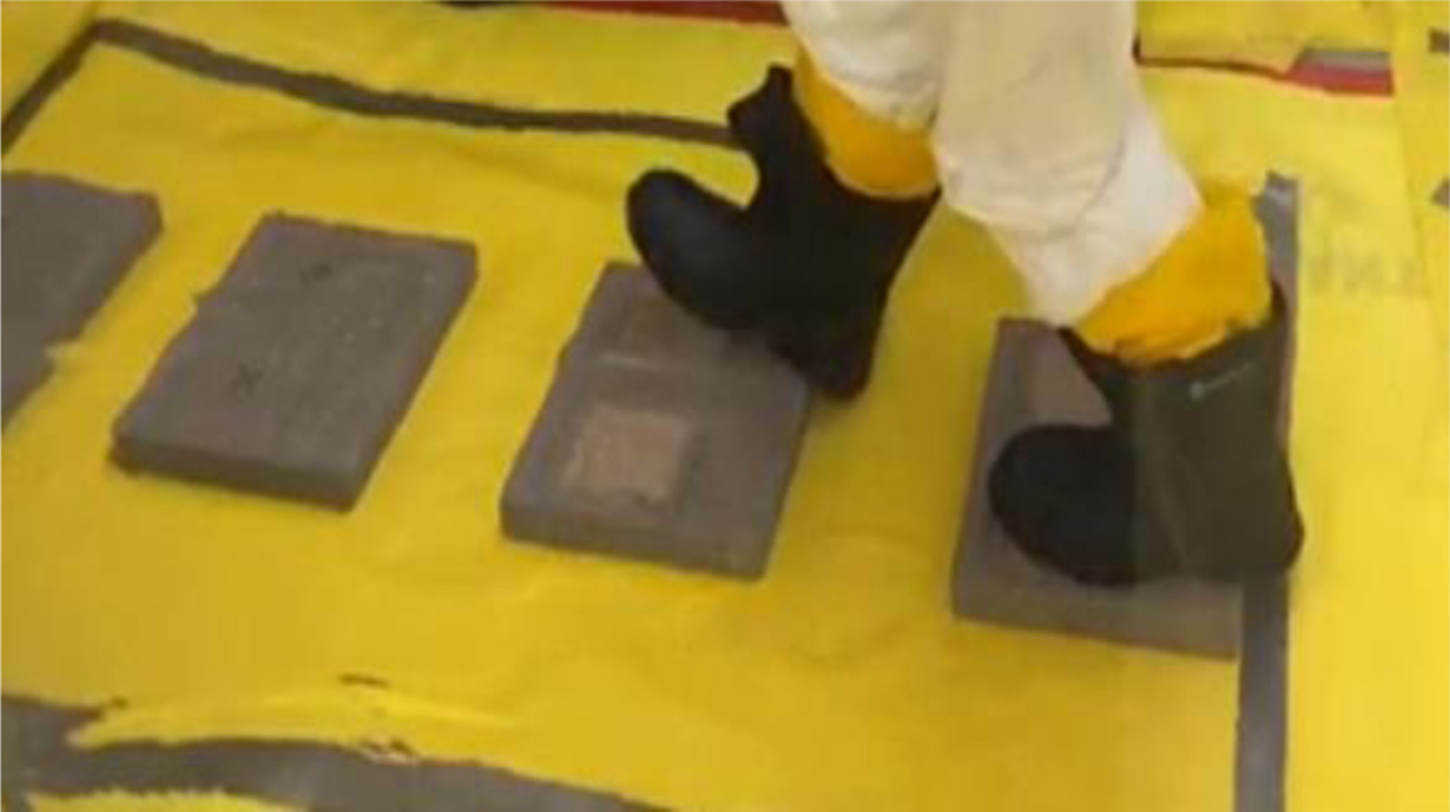
Straight-line Walking Experiment.

For foot traffic experiments, similar testing was performed with technicians wearing rubber boots (Item: Dunlog 86020, https://www.dunlopboots.com/, accessed on 9 April 2022) while walking across the testing surfaces. As with the simulated vehicle experiments, approximately 3 g of Sr-85 contaminated particles was applied to each testing surface with a surface area of 12.5 cm × 15 cm using a template. Four (4) contaminated surfaces were prepared for each testing surface as experiment controls. For stabilization technology testing experiments, the testing surfaces were dosed first with Sr-85 contaminated particles. After the surface was contaminated, a stabilization technology solution was sprayed onto the contaminated surfaces using a sprayer and allowed to dry (drying time ranged from overnight to 3 days, as the surfaces did not all dry completely overnight). CaCl_2_ and Soil_2_O solutions were also sprayed using a 2 gallon, multipurpose Scott’s sprayer with the cone spray setting. Phos-Chek MVP-Fx solution was sprayed using the same 0.5 gallon, multipurpose handheld sprayer as described above. Four (4) contaminated surfaces treated with each stabilization technology were prepared for each testing surface. The Sr-85 contaminated surfaces treated with/without stabilization technologies were tested first by walking across alternately placed contaminated surfaces, with one footstep on each surface, and subsequently onto previously uncontaminated surfaces (Figure 7 and Video S2). The boots were taken off and placed on a customized boot stand. The contaminated surfaces were measured for activity before and after walking. The activity of previously uncontaminated surfaces and Sr-85 transferred to the boots was measured.

#### 2.2.9. Circuit Path Walking Experiments

**Figure 8.**
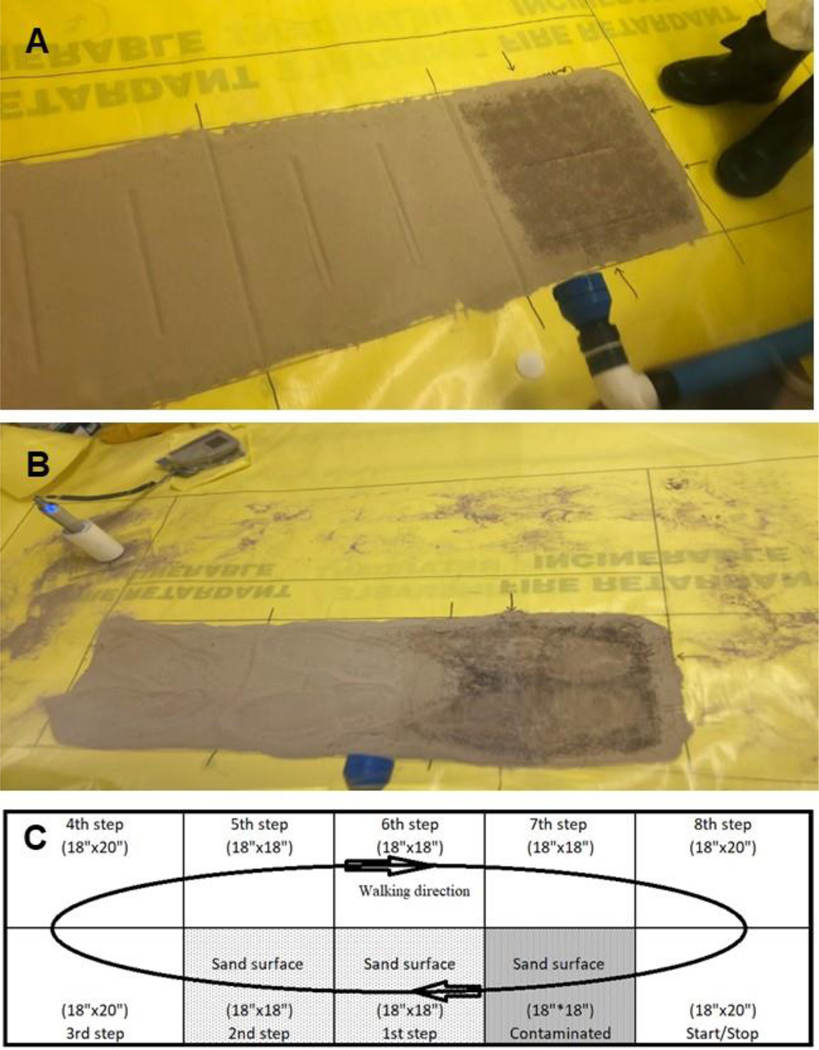
Appearance of Circuit Walking Path (**A**) Pre- and Post- (**B**) Walking for (**C**) Schematic Diagram for Circuit Walking Path Experiment. Panel (**B**) also depicts the use of the radiation detector for this type of experiment.

For the sand circuit walking path surfaces, the walking path included a contaminated sand surface (45 cm × 45 cm) connected with an uncontaminated surface (1st step and 2nd step including transfer surfaces, 45 cm × 90 cm) and non-sand surface (3rd step to 8th step and Start/Stop point). Figure 8 shows the post-walking condition of the control experiment and the walking path schematic diagram, and Video S3 shows an example experiment. To prepare the sand walking path, a premeasured amount of approximately 11 kg of loose sand was evenly distributed onto the defined area, including the contaminated area and the 1st step and 2nd step area. Approximately 40 g of Sr-85 contaminated particles were applied to the contaminated area of the walking path. To stabilize the contaminated surfaces, CaCl_2_, Phos-Chek MVP-Fx, and Soil_2_O solutions were sprayed using sprayers, as mentioned previously. One NSC experiment was contaminated with Sr-85 particles but was not sprayed with any stabilization technology. The other three experiments were performed similarly, but the contaminated surfaces were treated separately with the three stabilization technologies. The experiments were conducted to take the 1st step onto one-half of the 45 cm section of contaminated sand (with or without stabilization technologies). The next two steps were onto two (2) 45 cm sections of previously uncontaminated sand, followed by the remainder of the loop on the previously clean Herculite surface. Each time the walker stepped onto the contaminated surface, they alternated which foot they started with to keep the potential transfer as similar as possible. The walking path experiment was conducted for 5 continuous minutes (in the same direction) for approximately 36 passes.

**Figure 9.**
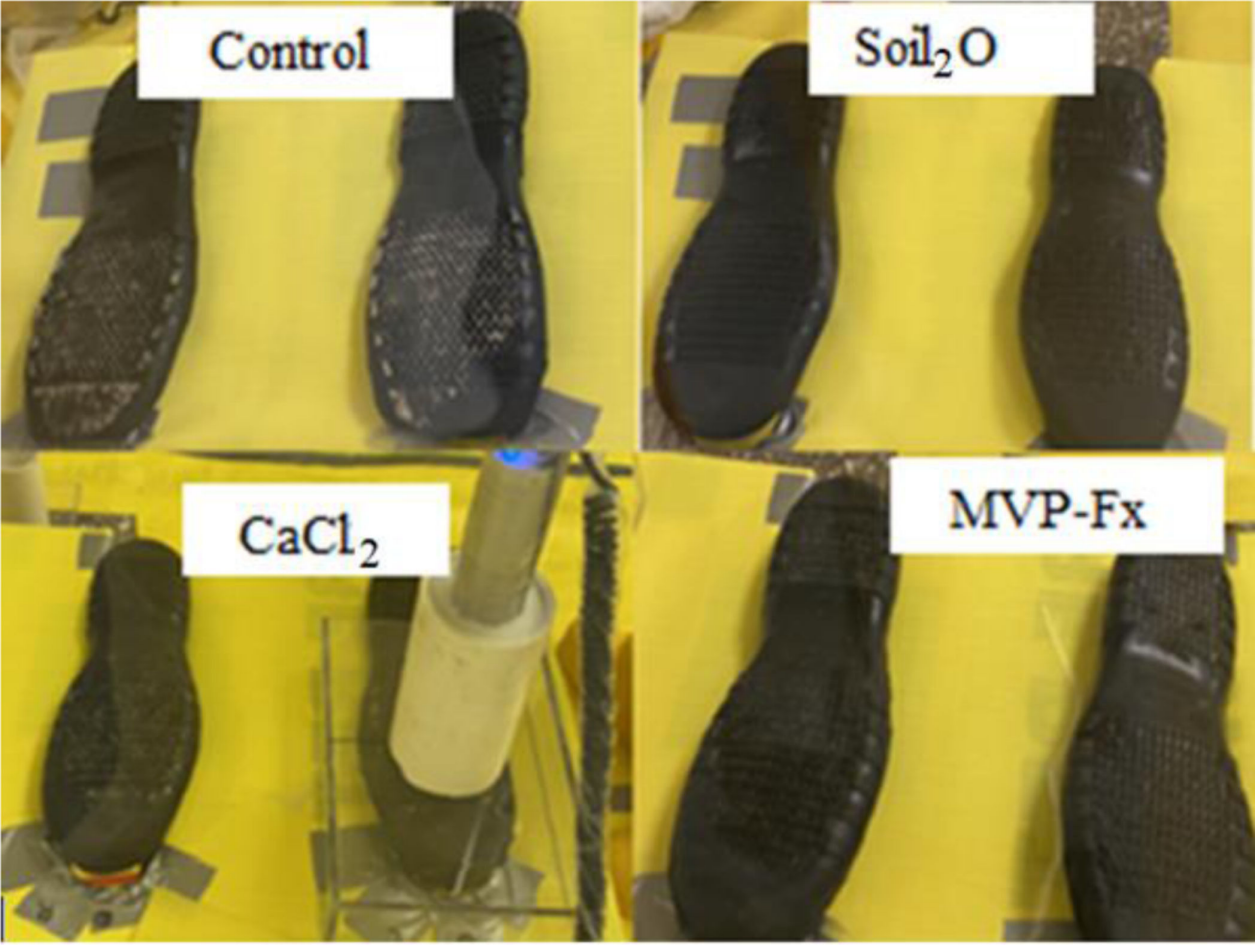
Customized stand for activity measurements from boots, shown for CaCl_2_. Also visible is residue on boots from contaminated asphalt without stabilization technology, indicated as the control, and with the labeled stabilization technologies. The use of the radiation detector is depicted for the CaCl_2_ stabilizer; its use was similar for the other stabilizers and the nonstabilized control.

The contaminated/stabilized surfaces were measured for activity before and after walking. The transferred surfaces and boots were also measured for the activity of Sr-85 to estimate the amount of transfer. All the measurements were accomplished by using an NaI spectrometer with customized stands made of Plexiglass^®^ to ensure a constant distance (1/2″) and geometry between the detector and the measured surfaces (Figure 9). The areas of each step where particles accumulated along the non-sand portion of the walking path were also measured for the activity of Sr-85 to further evaluate the transfer. A filter sample was placed on the floor at the edge of the contaminated soil surface to monitor the spread of Sr-85 contaminated particles, and an air sample with the sampling head deployed about 30 cm above the edge of the contaminated area was collected from each experiment using the same type of filters (47 mm FP-XM glass fiber filter, F&J Specialty Products, Inc., Ocala, FL, USA) to measure the resuspension of Sr-85 contaminated particles.

#### 2.2.10. Calculation of Percent Removal, Percent Transfer, and Percent Residual

The efficacy of stabilization technologies was evaluated through the calculation of percent removal (%R) after the application of stabilization technologies on different testing surfaces. The efficacy was also evaluated by calculating the percent transfer (%T) of activity from contaminated/stabilized surfaces to previously uncontaminated surfaces. These metrics were calculated using the following equations:

(1) $$\text{\%R}=(1-{\text{A}}_{\text{r}}/{\text{A}}_{\text{o}}\times 100\text{\%}$$

(2) $$\text{\%T}={\text{A}}_{\text{t}}/{\text{A}}_{\text{o}}\times 100\text{\%}$$

where ${\text{A}}_{\text{o}}$ is the activity from the contaminated/stabilized surface before vehicle-rolling or foot traffic, ${\text{A}}_{\text{r}}$ is the activity from the contaminated/stabilized surface after vehicle rolling or foot traffic, and ${\text{A}}_{\text{t}}$ is the activity from the previously uncontaminated surface after foot traffic or vehicle rolling experiments. The results were extrapolated by averaging to reflect the transfer of the whole surface.

The percent residual (%Res) remaining on the tires and boots after each experiment was calculated using the %T equation based on the residual activity of Sr-85 contaminated particles on tires/boots as the final activity and the activity of Sr-85 contaminated/stabilized testing surfaces as the original activity:

(3) $$\text{\%Res}={\text{A}}_{\text{Res}}/{\text{A}}_{\text{0}}\times 100\text{\%}$$

where ${\text{A}}_{\text{0}}$ is the activity from the contaminated/stabilized surface before vehicle-rolling or foot traffic, and ${\text{A}}_{\text{Res}}$ is the residual activity from the tires/boots. All the calculations were completed using the activity data collected from the experiments. Since each experiment lasted approximately 1.5 to 3 h, the data set for each experiment was used directly in the above calculations without any correction for radioactive decay. An independent group *t*-test at a 95% confidence interval was used to investigate differences in percent removal, transfer, and residual on tires/boots among different stabilization technologies.

## 3. Results and Discussion

The efficacy of the stabilization technologies selected was measured for each testing surface during simulated vehicle and foot traffic experiments. The %R of activity (from Sr-85 tagged particles) from the contaminated surface, %T of activity to a previously uncontaminated surface, and %Res activity remaining on the tire or boot after the vehicle and foot traffic experiment were calculated using the equations mentioned previously. All activities in the calculations were background-corrected.

### 3.1. Simulated Vehicle Experiments

The average %R, average %T, average %Res, and their corresponding standard deviation (SD) for each stabilization technology and testing surface are shown in Table 3. Four (4) replicates were conducted per stabilization technology and testing surface.

#### 3.1.1. Percent Removal from Contaminated Surfaces

**Figure 10.**
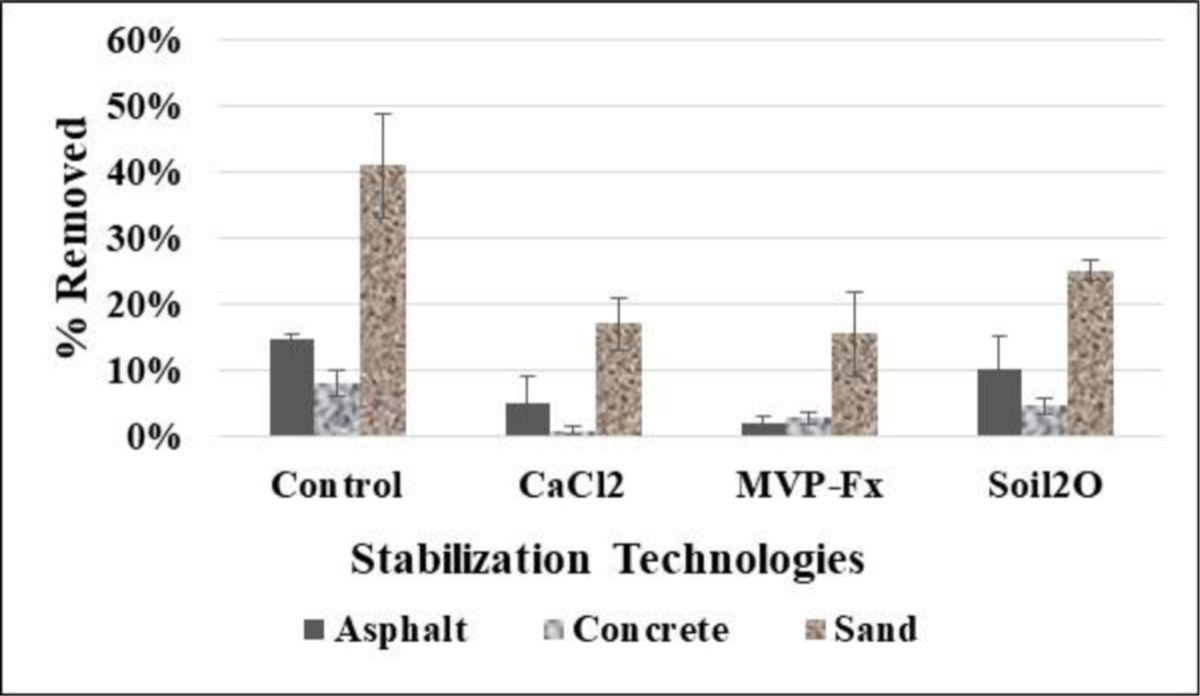
Percent Removal of Sr-85 Contaminated Particles for Vehicle Experiments.

Figure 10 shows the average %R of Sr-85 contaminated particles from the NSCs and the surfaces stabilized with CaCl_2_, Phos-Chek MVP-Fx, and Soil_2_O treated pavers. Table 4 lists the *p*-values from the *t*-tests performed. The *p*-values indicate the probability that the groupings of data were from the same population, i.e., statistically similar at the 95% level.

Generally, the potential for removal, indicated by the control %R, was highest for the sand surface, followed by asphalt and concrete. Interpreting the efficacy values in the figure in combination with the *p*-values reveals the following for specific surfaces.

##### Asphalt surfaces.

CaCl_2_ (5 ± 4 %R) and MVP-Fx (2 ± 1 %R) resulted in significantly decreased %R compared to the NSC (15 ± 1 %R), while Soil_2_O (10 ± 5 %R) did not. MVP-Fx (2 ± 1 %R) demonstrated significantly lower %R than Soil_2_O (10 ± 5 %R), but no significant difference from CaCl_2_ (5 ± 4 %R). CaCl_2_ (5 ± 4 %R) and Soil_2_O (10 ± 5 %R) generated %R values that were not significantly different from one another.

##### Concrete surfaces.

CaCl_2_ (0.9 ± 0.7 %R) and MVP-Fx (3 ± 1 %R) resulted in significantly decreased %R compared to the NSC (8 ± 2 %R), while Soil_2_O (5 ± 1 %R) did not. CaCl_2_ (0.9 ± 0.7 %R) demonstrated significantly lower %R than MVP-Fx (3 ± 1 %R) and Soil_2_O (5 ± 1 %R). MVP-Fx (3 ± 1 %R) demonstrated significantly lower %R than Soil_2_O (5 ± 1 %R), but significantly higher than CaCl_2_ (0.9 ± 0.7 %R). Soil_2_O (5 ± 1 %R) demonstrated %R values that were significantly higher than CaCl_2_ (0.9 ± 0.7 %R) and MVP-Fx (3 ± 1 %R).

##### Sand surfaces.

CaCl_2_ (17 ± 4 %R), MVP-Fx (16 ± 6 %R), and Soil_2_O (25 ± 2 %R) resulted in significantly decreased %R compared to the NSC (41 ± 8 %R). CaCl_2_ (17 ± 4 %R) and MVP-Fx (16 ± 6 %R) generated %R values that were not significantly different from one another. Soil_2_O (25 ± 2 %R) generated %R values that were significantly higher than CaCl_2_ (17 ± 4 %R) and MVP-Fx (16 ± 6 %R).

#### 3.1.2. Percent Transfer to Uncontaminated Surfaces

**Figure 11.**
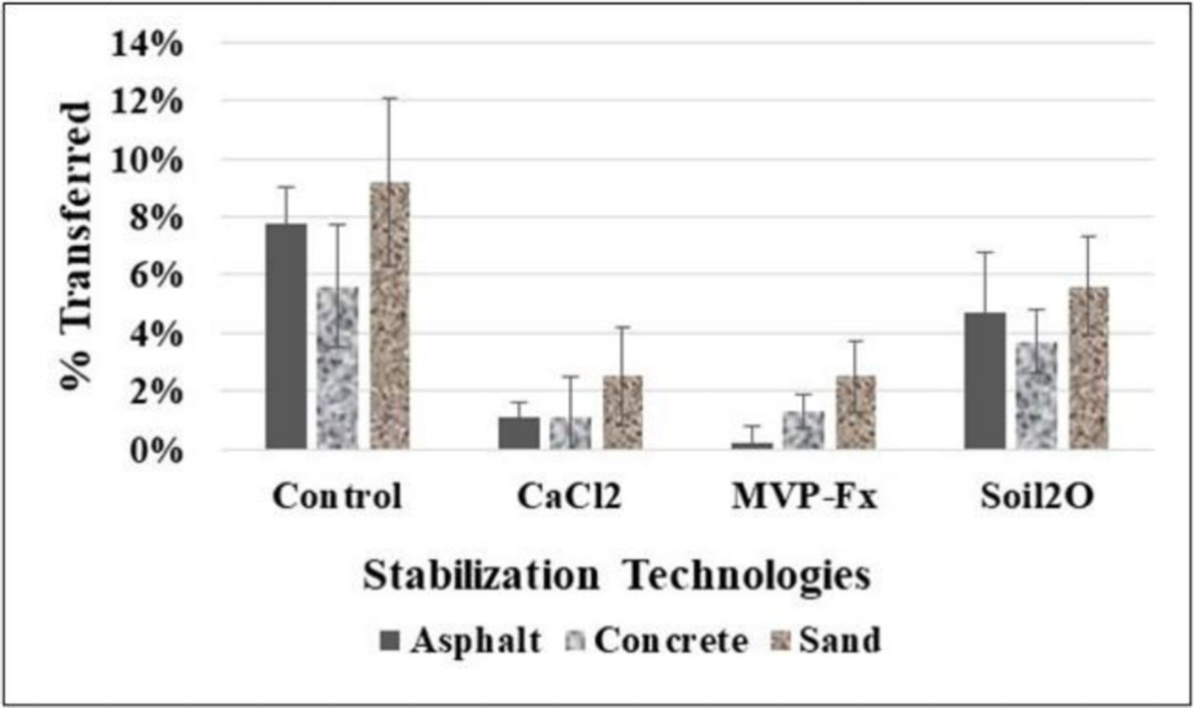
Percent Transfer of Sr-85 Contaminated Particles for Vehicle Experiments.

Figure 11 shows the average percent transfer of Sr-85-contaminated particles from the NSCs and from the surfaces that were stabilized with CaCl_2_, Phos-Chek MVP-Fx, and Soil_2_O treated pavers to pavers that were previously uncontaminated. Table 5 shows the *p*-values from the *t*-tests performed on the data. The *p*-values indicate the probability that the groupings of data were from the same population, i.e., statistically similar.

In general, the potential for transfer, indicated by the control %T, was similar for the three surfaces, as the average %T values ranged from approximately 6% to 9%. Interpreting the efficacy values in the figure in combination with the *p*-values from the table reveals the following for specific surfaces.

##### Asphalt.

CaCl_2_ (1 ± 1 %T), MVP-Fx (0.2 ± 0.6 %T), and Soil_2_O (5 ± 2 %T) resulted in significantly decreased %T compared to the NSC (8 ± 1 %T); the statistical power of the CaCl_2_ and MVP-Fx (*p* < 0.″01) were much higher than Soil_2_O (*p* = 0.04). CaCl_2_ (1 ± 1 %T) and MVP-Fx (0.2 ± 0.6 %T) generated %T values not significantly different from one another, and both produced %T values that were significantly less than Soil_2_O (5 ± 2 %T).

##### Concrete.

CaCl_2_ (1 ± 1 %T) and MVP-Fx (1 ± 1 %T) resulted in significantly decreased %R compared to the NSC (6 ± 2 %T), while Soil_2_O (4 ± 1 %T) did not. CaCl_2_ (1 ± 1 %T) and MVP-Fx (1 ± 1 %T) generated %T values that were not significantly different from one another, and both produced %T values that were significantly less than Soil_2_O (4 ± 1 %T).

##### Sand.

CaCl_2_ (3 ± 2 %T) and MVP-Fx (3 ± 1 %T) resulted in significantly decreased %R compared to the NSC (9 ± 3 %T), while Soil_2_O (6 ± 2 %T) did not. CaCl_2_ (3 ± 2 %T) and MVP-Fx (3 ± 1 %T) generated %T values that were not significantly different from one another, and both produced %T values that were significantly less than Soil_2_O (6 ± 2 %T).

#### 3.1.3. Percent Residual Activity on Simulated Vehicle Tires

**Figure 12.**
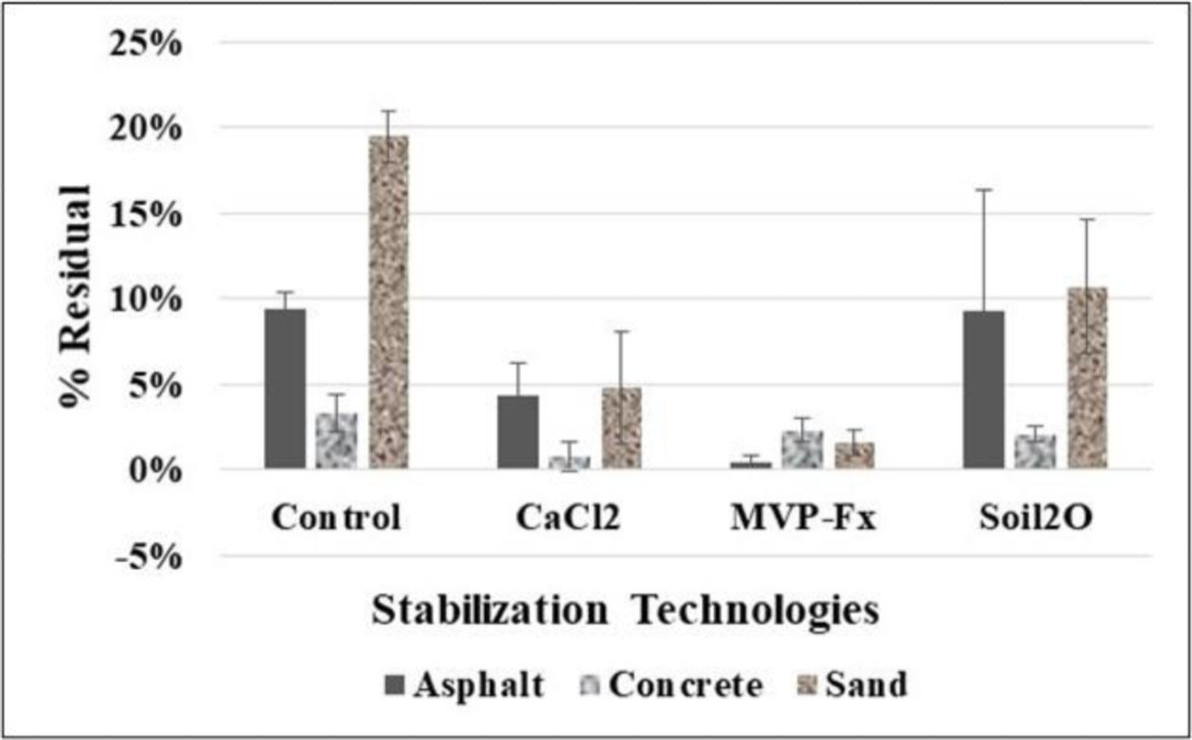
Percent Residual Activity of Sr-85 Contaminated Particles for Vehicle Experiments.

Figure 12 shows the average percent residual of Sr-85 contaminated particles that remained on simulated vehicle tires after each experiment, including the NSCs and surfaces stabilized with CaCl_2_, Phos-Chek MVP-Fx, and Soil_2_O. Table 6 shows the *p*-values from the *t*-tests performed on the data. The *p*-values indicate the probability that the groupings of data were from the same population, i.e., statistically similar.

The potential for residual, indicated by the control %Res, was highest for the sand surface, followed by asphalt and concrete. Interpreting the efficacy values in the figure in combination with the *p*-values from the table reveals the following for specific surfaces.

##### Asphalt.

CaCl_2_ (4 ± 2 %Res) and MVP-Fx (0.5 ± 0.4 %Res) resulted in significantly decreased %Res compared to the NSC (9 ± 1 %T), while Soil_2_O (9 ± 7 %Res) did not. MVP-Fx (0.5 ± 0.4 %Res) demonstrated significantly lower %R than CaCl_2_ (4 ± 2 %Res), and both of those produced less %Res than Soil_2_O (9 ± 7 %Res). However, the difference between MVP-Fx and Soil_2_O was not statistically significant (likely due to the relatively high variability of the Soil_2_O result).

##### Concrete.

Only CaCl_2_ (0.8 ± 0.9 %Res) resulted in significantly decreased %Res compared to the NSC (3 ± 1 %Res), while MVP-Fx (2 ± 1 %Res) and Soil_2_O (2 ± 1 %Res) did not. CaCl_2_ (0.8 ± 0.9 %Res) resulted in significantly lower %Res compared to both MVP-Fx (2 ± 0.7 %Res) and Soil_2_O (2 ± 0.5 %Res), which were not statistically different.

##### Sand.

CaCl_2_ (5 ± 3 %Res), MVP-Fx (2 ± 1 %Res), and Soil_2_O (11 ± 4 %Res) resulted in significantly decreased %Res compared to the NSC (20 ± 2 %Res). MVP-Fx (2 ± 1 %Res) demonstrated significantly lower %Res than Soil_2_O (11 ± 4 %Res) but was not significantly different from CaCl_2_ (5 ± 3 %Res). CaCl_2_ (5 ± 3 %Res) and Soil_2_O (11 ± 4 %Res) generated %Res that were not significantly different from one another.

#### 3.1.4. Simulated Vehicle Experimental Observations and Conclusions

Clearly, the sand surface provided the largest potential for transfer based on the sand having the largest %R and %Res during the NSC experiments. Overall, in most cases, the stabilization technologies provided improved stabilization compared to the specific NSC results, with CaCl_2_ and MVP-Fx generating lower average %R values and %T values in most cases. Both CaCl_2_ and MVP-Fx reduced the %R by at least a factor of 2 for all three surfaces tested, while Soil_2_O had a milder impact, demonstrating a factor of 0.4. However, there were a few instances in which one or more of the three stabilization technologies did not demonstrate a statistically significant improvement at the 95% confidence level. It is a limitation imposed by the radiological containment enclosure that vehicle speeds and vehicles representative of what would be used on a response location could not be studied. Airflow caused by the movement of vehicles and by the exhaust and fans coming from within the vehicle is extremely difficult to be simulated within radiological containment.

For data interpretation purposes, the %R and %T results are more reliable than the %Res results because of the nature of the measurement. Specifically, the %R and %T measurements were made by locating the detector probe in pre-marked positions on pavers with the same geometry with respect to the detector probe. For the %Res measurements, the detector probe was held near the tire, which had a curved contour both vertically and horizontally. In addition, the tires were measured at the center location of the expected tire contamination when the tire was oriented perpendicularly to the floor. Therefore, occasionally some particles, estimated < 10% in most cases, were observed to have fallen off the tire before the measurement was made.

### 3.2. Simulated Foot Traffic Experiments

Simulated foot traffic (i.e., walking) experiments were performed wearing boots with both straight-line walking on contaminated pavers and circuit walking on a path. Contaminated/stabilized pavers were measured for Sr-85 activity before and after walking to determine the %R. The transfer surfaces were measured for the activity of Sr-85 to evaluate the %T, and the boots were measured to determine the %Res on the boots after the experiment. The average %R, %T, %Res, and corresponding SD for each stabilization technology and testing surface are provided in Table 7. Four (4) replicates were conducted per stabilization technology and testing surface.

#### 3.2.1. Percent Removal from Contaminated Surfaces for Straight-Line Walking

**Figure 13.**
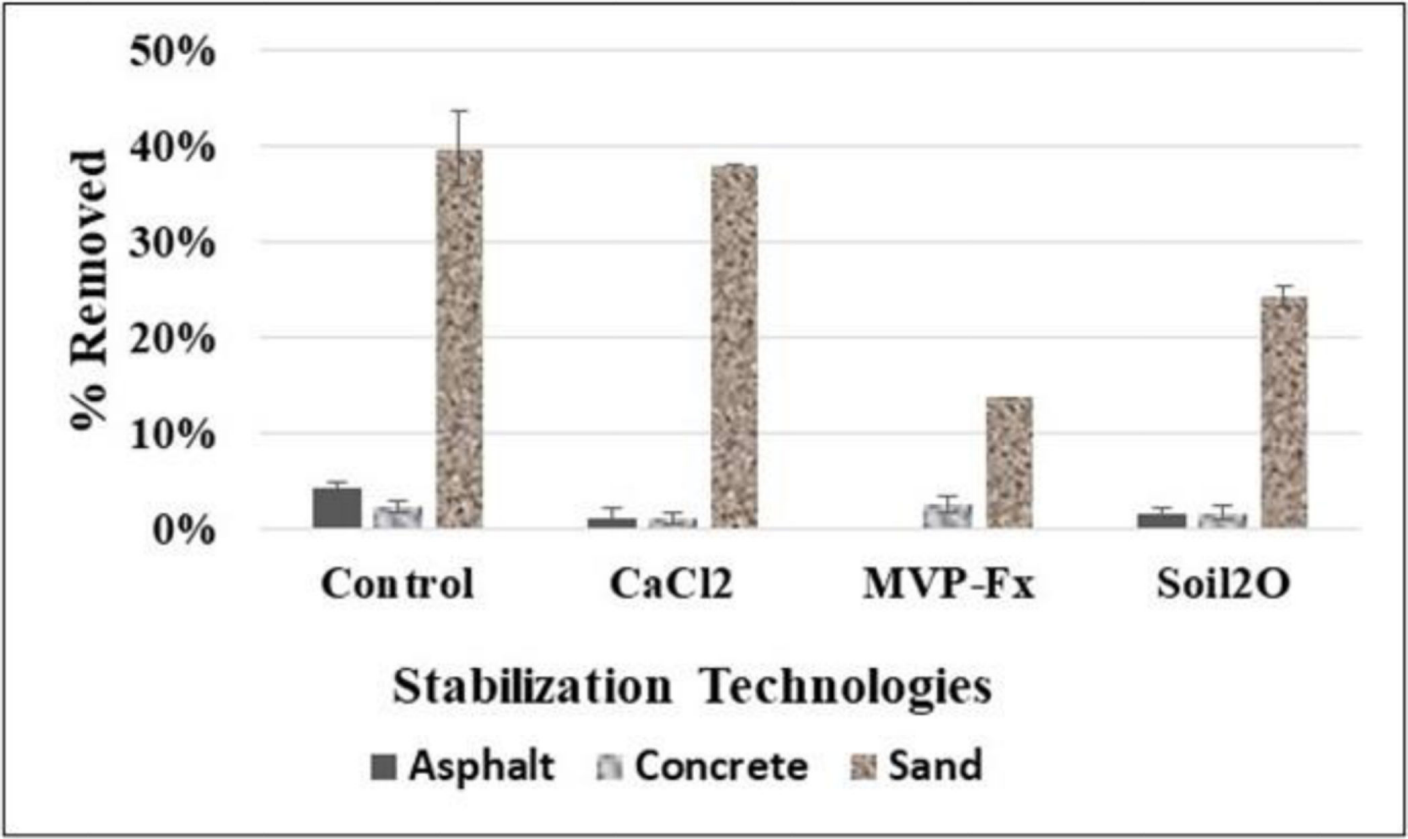
Percent Removal of Sr-85 Contaminated Particles for Straight-line Walking Experiments.

Figure 13 shows the average %R of Sr-85 contaminated particles from the NSCs and the surfaces stabilized with CaCl_2_, Phos-Chek MVP-Fx, and Soil_2_O treated pavers. Table 8 shows the *p*-values from the *t*-tests performed on the data. The *p*-values indicate the probability that the groupings of data were from the same population, i.e., statistically similar.

The potential for %R appears to be highest for the sand surfaces, followed by asphalt and concrete, as indicated by the control %R values. However, the sand results were determined from the 5 min continuous walking experiment, so the results are not exactly comparable. Interpreting the efficacy values in the figure in combination with the *p*-values from the table reveals the following for specific surfaces.

##### Asphalt.

CaCl_2_ (1 ± 1 %R), Soil_2_O (2 ± 1 %R) and MVP-Fx (Not Detected [ND]) resulted in significantly decreased %R compared to the NSC (4 ± 1 %R). MVP-Fx (ND) demonstrated significantly lower %R than Soil_2_O (2 ± 1 %R) and CaCl_2_ (1 ± 1 %R). CaCl_2_ (1 ± 1 %R) and Soil_2_O (2 ± 1 %R) had %R values not significantly different from one another.

##### Concrete.

Only CaCl_2_ (1 ± 1 %R) resulted in significantly decreased %R compared to the NSC (2 ± 1 %R) while MVP-Fx (3 ± 1 %R) and Soil_2_O (2 ± 1 %R) did not. CaCl_2_ (1 ± 1 %R) demonstrated significantly lower %R than MVP-Fx (3 ± 1 %R). CaCl_2_ (1 ± 1 %R) did not demonstrate significantly different %R from Soil_2_O (2 ± 1 %R). MVP-Fx (3 ± 1 %R) did not demonstrate significantly different %R from Soil_2_O (2 ± 1 %R).

##### Sand.

Note: There was a single experiment for each stabilization technology, and the SD was based on the right and left sides of the path [N = 2], so no *p*-values were calculated. MVP-Fx (14 ± 0.2 %R) and Soil_2_O (24 ± 0.9 %R) resulted in decreased %R compared to the NSC (40 ± 4 %R), while CaCl_2_ (38 ± 0.4 %R) did not. MVP-Fx (14 ± 0.2 %R) generated the lowest %R.

#### 3.2.2. Percent Transfer to Uncontaminated Surfaces for Straight-Line Walking

**Figure 14.**
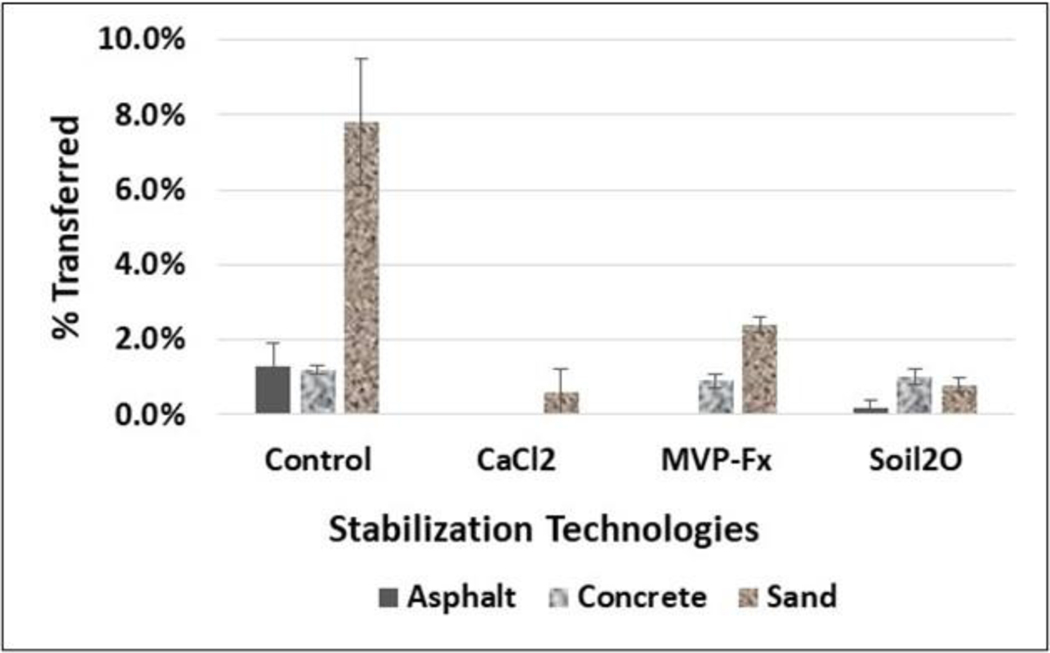
Percent Transfer of Sr-85 Contaminated Particles for Straight-line Walking Experiments.

Figure 14 shows the average %T of Sr-85 contaminated particles from the NSCs and from the surfaces stabilized with CaCl_2_, Phos-Chek MVP-Fx, and Soil_2_O treated pavers to pavers that were previously uncontaminated. Table 9 shows the *p*-values from the *t*-tests performed on the data. The *p*-values indicate the probability that the groupings of data were from the same population, i.e., statistically similar.

The potential for %T appeared to be highest for the sand surface, as evidenced by the control %T being more than four times larger than the other %T values. However, the sand results were determined from the 5 min continuous walking experiment, so the results are not exactly comparable. Interpreting the efficacy values in the figure in combination with the *p*-values from the table reveals the following for specific surfaces.

##### Asphalt.

CaCl_2_ (ND %T), MVP-Fx (ND %T), and Soil_2_O (0.2 ± 0.2 %T) resulted in significantly decreased %T compared to the NSC (1 ± 1 %T). CaCl_2_ (ND %T) and MVP-Fx (ND %T) generated %T values that were not significantly different from one another, and both produced %T values that were significantly less than Soil_2_O (0.2 ± 0.2 %T).

##### Concrete.

CaCl_2_ (ND %T) resulted in significantly decreased %T compared to the NSC (1 ± 0.1 %T), while MVP-Fx (0.9 ± 0.2 %T) and Soil_2_O (1 ± 0.2 %T) did not. Soil_2_O (1 ± 0.2 %T) and MVP-Fx (0.9 ± 0.2 %T) generated %T values that were not significantly different from one another, and both produced %T values that were significantly higher than CaCl_2_ (ND %T).

##### Sand.

Note: There was a single experiment for each stabilization technology, and the SD was based on the right and left sides of the path [N = 2], so no *p*-values were calculated. CaCl_2_ (0.6 ± 0.6 %T), MVP-Fx (2 ± 0.2 %T), and Soil_2_O (0.8 ± 0.2 %T) resulted in decreased %T compared to the NSC (8 ± 2%T).

### 3.3. Circuit Walking Path Results

**Figure 15.**
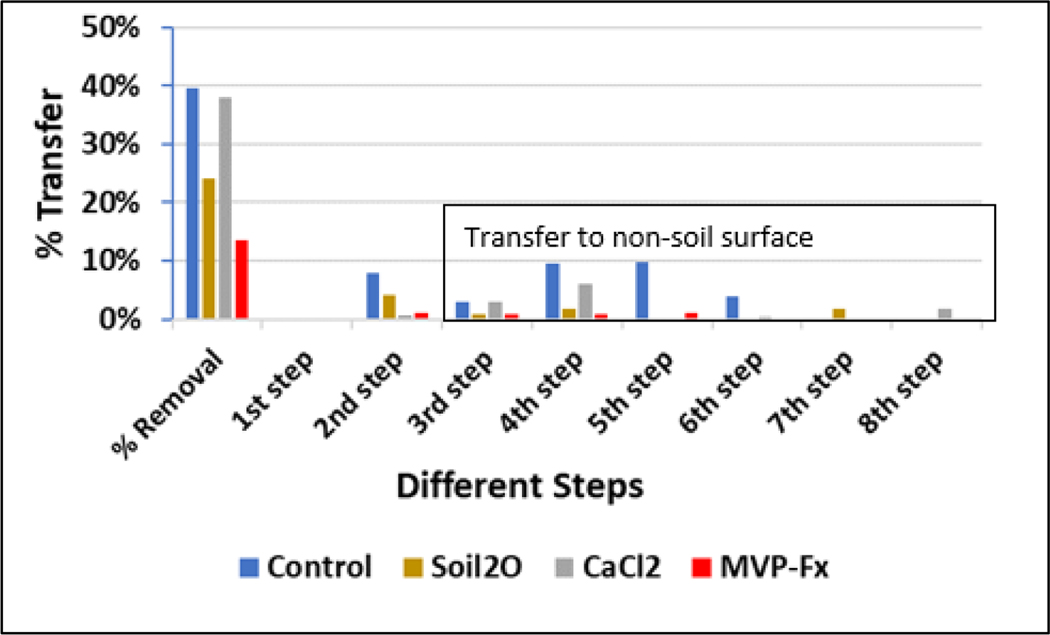
Percent Transfer for Walking Experiment on the Sand Walking Path.

For each stabilization technology, the average percent transfer of each step is provided in Table 10 and Figure 15. See Figure 8 for the NSC results for transferred sand and experimental schematic. The first step was always non-detect as the walker was always stepping from the noncontaminated area, after 8 steps on the non-sand surface, onto that area. The second step was taken from the contaminated area to the previously uncontaminated sand, so this area was expected to have had the highest degree of transfer. However, for the NSC and CaCl_2_, the fourth step (i.e., the footstep from the second step area) had the highest transfer. The CaCl_2_ stabilized sand seemed to form a crust that bound to the boot upon the first step, similar to the NSC as suggested by the CaCl_2_ %R, but the crust did not release from the boot until a step was made onto the non-sand surface. The third step area was expected to be relatively lower in transfer because the step was coming from the first step area. The measurable radioactivity was likely a product of kicking from the second step area or release from the boot upon stepping onto a different surface type. The radiological activities measured in the second step area, fourth step area, and sixth step area were generally higher, and more evident in the NSC than in the third, fifth, and seventh step area due to the more direct transfer of Sr-85 contaminated particles from the boot contact with those particles during every lap. The transfer of particles into the odd number step areas was due mostly to particles that had remained on the boot from at least the previous lap, or before.

#### 3.3.1. Percent Residual Activity on Boots from Circuit Walking

**Figure 16.**
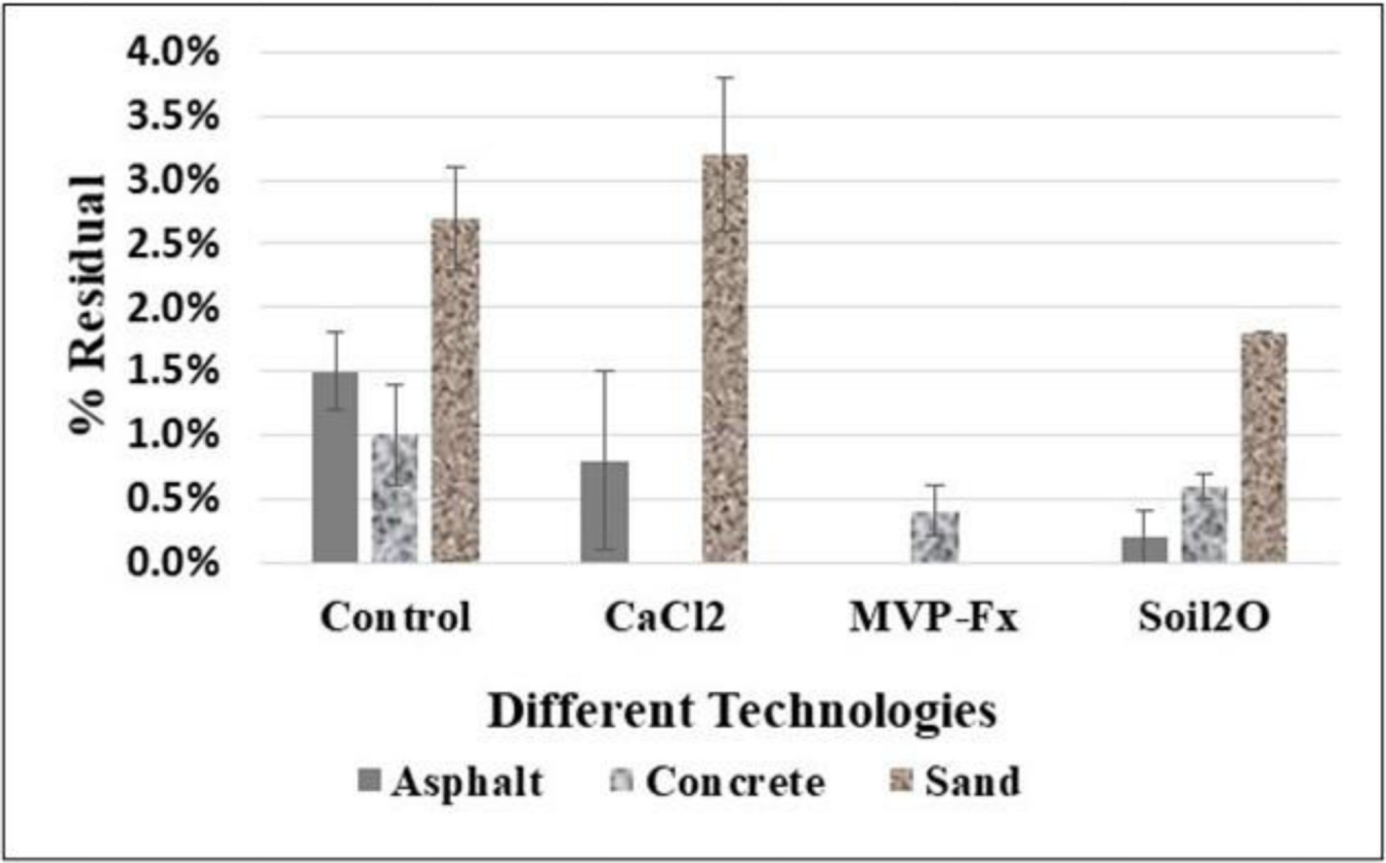
Percent Residual of Sr-85 Contaminated Particles from Foot Traffic Experiments.

Figure 16 shows the average %Res of Sr-85 contaminated particles that remained on boots after each experiment, including the NSCs and surfaces stabilized with CaCl_2_, Phos-Chek MVP-Fx, and Soil_2_O. Table 11 shows the p-values from the *t*-tests performed on the data. The *p*-values indicate the probability that the groupings of data were from the same population, i.e., statistically similar.

The potential for residual appeared to be highest for the sand surface, as indicated by the control %Res. However, the sand results were determined from the 5 min continuous walking experiment, so the results are not exactly comparable. Interpreting the efficacy values in the figure in combination with the *p*-values from the table reveals the following for specific surfaces.

##### Asphalt.

Soil_2_O (0.2 ± 0.2 %Res) and MVP-Fx (ND %Res) resulted in significantly decreased %Res compared to the NSC (2 ± 0.3 %T), while CaCl_2_ (0.8 ± 0.7 %Res) did not. MVP-Fx (ND %Res) demonstrated significantly lower %Res than CaCl_2_ (0.8 ± 0.7 %Res) and Soil_2_O (0.2 ± 0.2 %Res).

##### Concrete.

Only CaCl_2_ (ND %Res) resulted in significantly decreased %Res compared to the NSC (1 ± 0.4 %Res), while MVP-Fx (0.4 ± 0.2 %Res) and Soil_2_O (0.6 ± 0.1 %Res) did not, although MVP-Fx was close to being significantly different (*p* = 0.056). CaCl_2_ (ND %Res) resulted in significantly lower %Res compared to Soil_2_O (0.6 ± 0.1 %Res).

##### Sand.

Note: There was a single experiment for each stabilization technology, and the SD was based on the right and left sides of the path [N = 2], so no *p*-values were calculated. MVP-Fx (ND %Res) and Soil_2_O (2 ± 0 %Res) resulted in decreased %Res compared to the NSC (3 ± 0.4 %Res). CaCl_2_ (3 ± 0.6 %Res) generated higher average %Res than NSC (3 ± 0.4 %Res).

#### 3.3.2. Resuspension during Circuit Walking

A filter sample was deployed near the edge of the transition area (from Sr-85 contaminated sand surface to uncontaminated loose sand area) for each walking experiment on the sand walking path to monitor the spread of Sr-85 contaminated particles adjacent to the contaminated area. During the experiments, there were no visible particles transferred (kicked or dragged, etc.) over to the filter, and the measurements for each experiment were extremely close to the background measurements. The NSC sample produced a non-detect result, while each stabilization technology experiment produced less than 4 counts per second (cps), indicating a minimal presence of radiological material.

For the air sampler (5 cfm flow) set up approximately 30 cm above the ground near the contaminated area to collect an air sample for each sand walking path experiment, radiological activity was detected only from the air samples collected during the NSC experiment (2.8 × 10^−10^ μCi/mL) and the Soil_2_O stabilization technology (9.7 × 10^−11^ μCi/mL). These values are two to three orders of magnitude less than the derived air concentration (DAC) value (6 × 10^−7^ μCi/mL for Sr-85 particles potentially retained for years after exposure) listed in the US regulations for standards for radiation protection [19]. The DAC is defined as the concentration of a given radionuclide in the air which, if breathed for a working year of 2000 h under conditions of light work (1.2 cubic meters of air per hour), results in an intake of one annual limit on intake. The DAC values are presented for information only because they are intended to control chronic occupational exposure and are calculated based on a semi-infinite cloud of uniform concentration. This is not necessarily the exposure these experiments are intended to simulate, so the relationship between DAC and the experimental results may differ in an actual incident.

Particles containing approximately 80 μCi were placed on the surface prior to the walking experiment. For the NSC experiment, approximately 2 × 10^−4^ μCi was measured on the air sample, and for the Soil_2_O experiment, approximately 7 × 10^−5^ μCi was measured on the air sample. The ratio of measured activity in the air to the activity available on the ground was 2.5 × 10^−6^ and 9 × 10^−7^ for the NSC and Soil_2_O experiments, respectively. Albeit slight, the Soil_2_O experiments indicated a decreased availability of particles for inhalation. Neither air sample collected during the CaCl_2_ and Phos-Chek MVP-Fx experiments generated detectable radiological activity, indicating they also functioned to decrease the availability of particles. During each simulated vehicle and foot traffic experiments (and all the experimental preparatory work, including particle contamination), two air samples (3 cfm flow) were collected in the general work area of the containment tent. The average concentration of these samples was 4.9 × 10^−13^ μCi/mL, approximately six orders of magnitude less than the DAC.

#### 3.3.3. Foot Traffic Experimental Observations and Conclusions

Similar to the vehicle experiments, the individual results can be viewed in the context of the statistical result summaries above. For the asphalt and concrete experiments, less than 5% of the particles were removed from the NSC experiments, whereas during the walking path experiment, almost 40% of the particles were removed. In most cases, the stabilization technologies provided improved stabilization compared to the surface-type-specific NSC experiment results. However, there were a few instances in which one or more of the three stabilization technologies did not demonstrate statistically significant improvement or had high removals even with the stabilizer. This often occurred for sand which had the highest NSC, suggesting sand is a less stable surface to begin with, which is arguably intuitive when compared to more solid surfaces like concrete and asphalt. This also tended to occur for the CaCl_2_, which tended to form a crust on the boot, increasing the removal from the surface of radioactivity contained in the crust.

Also, similar to the simulated vehicle experiments, the %R and %T results are more reliable than the %Res results because of the nature of the measurement. The %R and %T measurements were made by locating the detector probe in pre-marked positions on pavers with the same geometry with respect to the detector probe. For the %Res measurements, the detector probe was held near the boots, which had an uneven contour, and the sand would bind to somewhat different locations depending on the experiment.

## 4. Conclusions

Removal percentages, with or without stabilizers, were higher from the soil than from asphalt or concrete. Therefore, stabilization of contaminated soils surfaces can represent an important goal for stabilizer application. The data and statistical analysis presented can aid in selecting appropriate stabilizers because they indicate the potential for stabilization techniques to enable operations in contaminated areas. In addition, the process of performing the studies revealed numerous operational details and observations for each of the stabilization technologies pertaining to application, surface material interactions, health and safety concerns, and estimated cost. These are summarized in Tables S1–S3 for each of the three stabilizers.

Related to the selection of the stabilizer at a particular site, this study also suggests which of the several measures of contaminant spread may be most useful to determine from future studies, whether on the laboratory, pilot, or field scale. Namely, in general, percent removal from surface (%R) and percent transferred from contaminated to an uncontaminated surface (%T) seemed more useful in performing statistical comparisons between experiments. The value of the percent residue left on the tire or boot (%Res) was often limited by the ability to reliably measure the radioactivity on the tire or boot, often for practical reasons.

Many conclusions from the specific types of experiments are discussed above, but further refinement of this information would result from field-scale studies, which better represent all the forces that stabilized particles are subjected to. For instance, based on the logistical constraints for walking experiments in the contamination enclosure tent, it was not possible to extensively vary operationally important variables like worker mass, walking speed, stride length, boot design and size, and other operator-specific variables that could affect the physical stresses that walking imparts upon the stabilized radioactivity and its ultimate resuspension. Likewise, for the vehicle experiments, it was impossible to achieve realistic vehicle speeds in the radiological containment enclosure. Other variables could include tire tread pattern and acceleration rate, which may be even more important as electric, high torque vehicles, especially private ones, become more common. Electric vehicles may result in higher resuspension due to the higher mass of their batteries [20,21]. Further, differently shaped vehicles will have different aerodynamics and the resulting potential for air disturbance. Airflow caused by the movement of vehicles and by the exhaust and fans issuing from within the vehicle is extremely difficult to be simulated within a radiological containment tent.

Testing additional stabilization agents for resuspension resulting from foot and vehicle traffic is warranted. For instance, in a study of wind-driven suspension, different saltwater compositions yielded somewhat different results, with some results dependent on the nature of the physical stresses on the stabilized particles [15]. Interestingly, this dependency did not exist for one saltwater mixture, namely that from an aqueous solution from the Dead Sea. Such dependencies can introduce uncertainty into operational decisions. Thus, in devising studies related to foot and vehicle traffic resuspension, achieving the desirable goal of seeking stabilizing technologies independent of the surface seems promising.

## Supplementary Material

Supplementary Information

Video S1

Video S2

Video S3

## Figures and Tables

**Table 1.** Particle size distribution of wetted and nonwetted simulated fallout material.

Particle Size Analysis	Wetted	Nonwetted
Clay (%)	24.00	28.00
Silt (%)	59.90	56.70
Sand (%)	16.10	15.30
Organic Matter (%)	0.43	0.25
Fine Gravel (%)	0	0
**Sand Fractions (%)**		
1 mm	0.10	0.00
0.25 mm	1.10	0.00
0.15 mm	0.60	0.20
0.05 mm	14.30	15.10

**Table 2.** Particle Size Distribution of Different Soils.

Particle Size Analysis	Israel Soil Samples		Experimental Bulk Soil	
	Ze’elim	Rotem	Ohio Mulch	Garick
Clay (%)	4	2	1.5	2
Silt (%)	4	1.2	1.6	0.8
Sand (%)	92	96.8	74.4	97.2
Organic Matter (%)	0.08	0	0.87	0.05
Fine Gravel (%)	0	0	22.5	0
**Sand Fractions (%)**				
1 mm	0.1	1	12.6	1.4
0.25 mm	28.9	87.3	59.6	74
0.15 mm	10.6	5.8	1.4	12.3
0.05 mm	52.4	2.7	0.08	9.5
**Texture Class**	Sand	Sand	Sand	Sand

**Table 3.** Efficacy of stabilization technologies during simulated vehicle experiments, expressed as average ± standard deviation.

Test Surface	Stabilizaiton Technology	Initial CPS (Average)	Removal (%R)	Transferred (%T)	Residual (%R)
Asphalt	Control	1497	15 ± 1	8±1	9±1
	CaCl_2_	1089	5 ± 4	1±1	4±2
	MVP-Fx	1292	2 ± 1	0.2 ± 0.6	0.5 ± 0.4
	Soil_2_O	1210	10 ± 5	5±2	9±7
Concrete	Control	1773	7±3	5±2	3±1
	CaCl_2_	1219	0.9 ± 0.7	1±1	0.8 ± 0.9
	MVP-Fx	1346	3 ± 1	1±1	2±1
	Soil_2_O	1382	5 ± 1	4±1	2±1
Sand	Control	1363	41 ± 8	9±3	20 ± 2
	CaCl2	1303	17 ± 4	3±2	5±3
	MVP-Fx	1312	16 ± 6	3±1	2±1
	Soil_2_O	1325	25 ± 2	6±2	11 ± 4

**Table 4.** Statistical results (*p*-values) for simulated vehicle experiments percent removal. Gray shading indicates a significant difference (*p* < 0.05).

Surface	Stab. Tech.	CaCl_2_	MVP-Fx	Soil_2_O
Asphalt	Control	0.0172	<0.0001	0.1764
	CaCl_2_		0.2535	0.1655
	MVP-Fx			0.0475
Concrete	Control	0.0255	0.0394	0.2081
	CaCl_2_		0.0133	0.0010
	MVP-Fx			0.0322
Sand	Control	0.0017	0.0023	0.0251
	CaCl_2_		0.6876	0.0101
	MVP-Fx			0.0256

**Table 5.** Statistical results (*p*-values) for simulated vehicle experiments percent transfer. Gray shading indicates a significant difference (*p* < 0.05).

Surfaces	Stab. Tech.	CaCl_2_	MVP-Fx	Soil_2_O
Asphalt	Control	<0.0001	<0.0001	0.0444
	CaCl_2_		0.0802	0.0160
	MVP-Fx			0.0065
Concrete	Control	0.0139	0.0072	0.2285
	CaCl_2_		0.8527	0.0281
	MVP-Fx			0.0073
Sand	Control	0.0070	0.0055	0.0759
	CaCl_2_		0.9631	0.0411
	MVP-Fx			0.0277

**Table 6.** Statistical results (*p*-values) for simulated vehicle experiments percent residual. Gray shading indicates a significant difference (*p* < 0.05).

Surfaces	Stab. Tech.	CaCl_2_	MVP-Fx	Soil_2_O
Asphalt	Control	0.0036	<0.0001	0.9709
	CaCl_2_		0.0267	0.2282
	MVP-Fx			0.0907
Concrete	Control	0.0089	0.1401	0.0743
	CaCl_2_		0.0388	0.0404
	MVP-Fx			0.7378
Sand	Control	0.0002	<0.0001	0.0056
	CaCl_2_		0.1503	0.0601
	MVP-Fx			0.0169

**Table 7.** Efficacy of stabilization technologies for simulated foot traffic experiments, expressed as average ± standard deviation.

Test Surface	Stabilizaiton Technology	Initial CPS (Average)	Removal (%R)	Transferred (%T)	Residual (%R)
Asphalt	Control	1918	4 ± 1	1 ± 0.6	2 ± 0.3
	CaCl_2_	1283	1 ± 1	ND	0.8 ± 0.7
	MVP-Fx	1671	ND	ND	ND
	Soil_2_O	1688	2 ± 1	0.2 ± 0.2	0.2 ± 0.2
Concrete	Control	1869	2±1	1 ± 0.1	1 ± 0.4
	CaCl_2_	1794	1 ± 1	ND	ND
	MVP-Fx	1685	3 ± 1	0.9 ± 0.2	0.4 ± 0.2
	Soil_2_O	1575	2 ± 1	1 ± 0.2	0.6 ± 0.1
Sand	Control	3401	40 ± 4	8±2	3.0 ± 0.4
	CaCl_2_	2683	38.0 ± 0.4	0.6 ± 0.6	3±1
	MVP-Fx	2544	14.0 ± 0.2	2.0 ± 0.2	0±0
	Soil_2_O	2968	24.0 ± 0.9	0.8 ± 0.2	2±0

**Table 8.** Statistical results (*p*-values) for foot traffic experiment percent removal. Gray shading indicates a significant difference (*p* < 0.05).

Surfaces	Stab. Tech.	CaCl_2_	MVP-Fx	Soil_2_O
Asphalt	Control	0.0024	<0.0001	0.0009
	CaCl_2_		0.0366	0.5490
	MVP-Fx			0.0027
Concrete	Control	0.0429	0.5701	0.2801
	CaCl_2_		0.0230	0.3127
	MVP-Fx			0.1435

**Table 9.** Statistical results (*p*-values) for foot traffic experiments percent transfer. Gray shading indicates a significant difference (*p* < 0.05).

Surfaces	Stab. Tech.	CaCl_2_	MVP-Fx	Soil_2_O
Asphalt	Control	0.0035	0.0121	0.0108
	CaCl_2_		0.7594	0.0422
	MVP-Fx			0.0010
Concrete	Control	<0.0001	0.0407	0.1677
	CaCl_2_		<0.0001	<0.0001
	MVP-Fx			0.4502

**Table 10.** Percent transfer of walking experiments to sand and non-sand.

Step Number	Control	Soil_2_O	CaCl_2_	MVP-Fx
1	ND ^1^	ND	ND	ND
2	8%	4%	0.6%	1%
3	3%	0.8%	3%	0.8%
4	10%	2%	6%	0.9%
5	1%	0.2%	NC	1%
6	4%	ND	0.3%	ND
7	ND	2%	ND	ND
8	NC ^2^	ND	2%	ND

1 ND = Not detected.

2 NC = Not collected because no particles were deposited.

**Table 11.** Statistical results (*p*-values) for foot traffic experiments percent residual. Gray shading indicates a significant difference (*p* < 0.05).

Surfaces	Stab. Tech.	CaCl_2_	MVP-Fx	Soil_2_O
Asphalt	Control	0.1143	<0.0001	0.0004
	CaCl_2_		0.0481	0.1662
	MVP-Fx			0.0023
Concrete	Control	0.0120	0.0553	0.1460
	CaCl_2_		0.0799	0.0157
	MVP-Fx			0.1769

## Data Availability

All data is found within the tables of this manuscript.

## References

[1] Kaminski MD; Lee SD; Magnuson M. Wide-area decontamination in an urban environment after radiological dispersion: A review and perspectives. J. Hazard. Mater 2016, 305, 67–86. [CrossRef] [PubMed] 26642448 10.1016/j.jhazmat.2015.11.014

[2] Amato F; Querol X; Johansson C; Nagl C; Alastuey A. A review on the effectiveness of street sweeping, washing and dust suppressants as urban PM control methods. Sci. Total Environ. 2010, 408, 3070–3084. 20488509 10.1016/j.scitotenv.2010.04.025

[3] Gulia S; Goyal P; Goyal S; Kumar R. Re-suspension of road dust: Contribution, assessment and control through dust suppressants—A review. Int. J. Environ. Sci. Technol 2019, 16, 1717–1728.

[4] Norman M; Johansson C. Studies of some measures to reduce road dust emissions from paved roads in Scandinavia. Atmos. Environ 2006, 40, 6154–6164.

[5] Saito H; Sutton M; Zhao P; Lee SD; Magnuson M. Review of technologies for preventing secondary transport of soluble and particulate radiological contamination from roadways, roadside vegetation, and adjacent soils. Environ. Adv 2020, 1, 100003. 10.1016/j.envadv.2020.100003 PMC10208302 37229463

[6] Xu L; Pei Z. Preparation and optimization of a novel dust suppressant for construction sites. J. Mater. Civ. Eng 2017, 29, 04017051.

[7] Zhan Q; Qian C; Yi H. Microbial-induced mineralization and cementation of fugitive dust and engineering application. Constr. Build. Mater 2016, 121, 437–444.

[8] Zhang B; Wang Y; Zhao X; Cao L; Tong R. Effectiveness of road dust suppressants: Insights from particulate matter-related health damage. Environ. Geochem. Health 2021, 43, 4139–4162. [CrossRef] [PubMed] 33778916 10.1007/s10653-021-00866-6

[9] Parvej S; Naik DL; Sajid HU; Kiran R; Huang Y; Thanki N. Fugitive Dust Suppression in Unpaved Roads: State of the Art Research Review. Sustainability 2021, 13, 2399.

[10] Tsogt B; Oh S-Y Preparations and application of dust suppressants from biomass-based materials. J. Air Waste Manag. Assoc 2021, 71, 1386–1396. [CrossRef] [PubMed] 34128771 10.1080/10962247.2021.1942320

[11] Stallworth AM; Chase EH; McDevitt B; Marak KK; Freedman MA; Wilson RT; Burgos WD; Warner NR Efficacy of oil and gas produced water as a dust suppressant. Sci. Total Environ. 2021, 799, 149347. [CrossRef] [PubMed] 34426301 10.1016/j.scitotenv.2021.149347 PMC8530883

[12] Li S; Zhao B; Lin H; Shuang H; Kong X; Yang E. Review and prospects of surfactant-enhanced spray dust suppression: Mechanisms and effectiveness. Process Saf. Environ. Prot 2021, 154, 410–424.

[13] USEPA. Wide Area Stabilization of Radiological Particulate Contamination. EPA/600/R-616/067. 2017. Available online: https://cfpub.epa.gov/si/si_public_record_report.cfm?Lab=NHSRC&dirEntryId=335602 (accessed on 9 April 2022).

[14] USEPA. Technical Report for the Demonstration of Wide Area Radiological Decontamination and Mitigation Technologies for Building Structures and Vehicles. EPA/6″/R-616/019. 2016. Available online: https://cfpub.epa.gov/si/si_public_record_report.cfm?Lab=NHSRC&dirEntryId=312072 (accessed on 9 April 2022).

[15] Raveh-Amit H; Sharon A; Katra I; Stilman T; Serre S; Archer J; Magnuson M. Limiting Wind-Induced Resuspension of Radioactively Contaminated Particles to Enhance First Responder, Early Phase Worker and Public Safety—Part 1. Appl. Sci 2022, 12, 2463. 10.3390/app12052463 PMC10494888 37701659

[16] IWTSD. Irregular Warfare Technical Support Directorate. 2022. Available online: https://www.cttso.gov (accessed on 9 April 2022).

[17] Clark DE; Cobbin WC Removal Effectiveness of Simulated Dry Fallout from Paved Areas by Motorized and Vacuumized Street Sweepers; Naval Radiological Defense Lab: San Francisco, CA, USA, 1963. Available online: https://apps.dtic.mil/sti/pdfs/AD0456495.pdf (accessed on 9 April 2022).

[18] Wiltshire LL; Owen LW Three Tests of Firehosing Technique and Equipment for the Removal of Fallout from Asphalt Streets and Roofing Materials; Naval Radiological Defense Lab: San Francisco, CA, USA, 1966. Available online: https://apps.dtic.mil/sti/pdfs/AD0640491.pdf (accessed on 9 April 2022).

[19] NRC. Appendix B to Part 20—Annual Limits on Intake (ALIs) and Derived Air Concentrations (DACs) of Radionuclides for Occupational Exposure; Effluent Concentrations; Concentrations for Release to Sewerage. 2021. Available online: https://www.nrc.gov/reading-rm/doc-collections/cfr/part020/part020-appb.html (accessed on 9 April 2022).

[20] Alam MS; Hyde B; Duffy P; McNabola A. Analysing the Co-Benefits of transport fleet and fuel policies in reducing PM2.5 and CO2 emissions. J. Clean. Prod 2018, 172, 623–634.

[21] Piscitello A; Bianco C; Casasso A; Sethi R. Non-exhaust traffic emissions: Sources, characterization, and mitigation measures. Sci. Total Environ. 2021, 766, 144440. [CrossRef] [PubMed] 33421784 10.1016/j.scitotenv.2020.144440

