# Supplementary material for: Part 2: Stabilization/Containment of Radiological Particle Contamination to Enhance First Responder, Early Phase Worker, and Public Safety
Source: Appl Sci (Basel). Author manuscript; Available in PMC 2023 Oct 17. (PMC10581405; doi:10.3390/app12083861)
Supplement: Supplementary Information [file NIHMS1920368-supplement-Supplementary_Information.pdf]

Supplemental Information

Part 2. Stabilization/Containment of Radiological Particle Contamination to Enhance First Responder, Early Phase Worker, and Public Safety

Matthew Magnuson [1,*], Terry Stilman [2], Shannon Serre [3], John Archer [1], Ryan James [4], Xiaoyan Xia [4], Mitchell Lawrence [4], Erin Tamargo [5], Hadas Raveh-Amit [6] and Avi Sharon [7]

[1] EPA Office of Research and Development, Homeland Security Materials and Management Division, 26 W Martin Luther King Dr, Cincinnati, OH 45268, USA; archer.john@epa.gov
[2] EPA Region 4, 61 Forsyth St, SW, Atlanta, GA 30303, USA; stilman.terry@epa.gov
[3] EPA Office of Land and Emergency Management, Consequence Management Advisory Division, WJC-N, Washington, DC 20002, USA; serre.shannon@epa.gov
[4] Battelle Memorial Institute, 505 King Ave, Columbus, OH 43201, USA; jamesr@battelle.org (R.J.); xia@battelle.org (X.X.); lawrencem1@battelle.org (M.L.)
[5] Irregular Warfare Technical Support Directorate, Alexandria, VA 22350, USA; erin.tamargo@iwtsd.gov
[6] Department of Chemistry, Nuclear Research Centre Negev, P.O. Box 9001, Beer Sheva 8419000, Israel; hadasa@nrcn.gov.il
[7] Environmental Research Unit, Nuclear Research Centre Negev, P.O. Box 9001, Beer Sheva 8419000, Israel; avish@nrcn.gov.il
* Correspondence: magnuson.matthew@epa.gov

Video S1. Simulated Vehicle Experiment Example

Vehicle expt.mp4

Video S2. Straight-line Walking Experiment Example

Stepwise expt.mp4

Video S3. Circuit Walking Path Experiment Example

Walking Path expt.mp4

Table S1. $CaCl_2$ Operational Information.

Parameters	Operational Information
Application	**$CaCl_2$** was prepared as a dilute aqueous solution (with a viscosity similar to water) and applied using a 2-gallon garden sprayer as described above. The aqueous solution was applied gently not to disturb the particles on the surface until the particles were fully wetted, but before pooling on the surface occurred. The stabilization technologies were allowed to dry to the touch (at least overnight; 2-3 days for asphalt) before experiments were performed.
Surface interactions	**Asphalt:** $CaCl_2$ was applied as described above and as the surfaces dried, the contaminated particles did not always dry to a lighter color compared to when they were wetted. Some of the contaminated particles maintained a darker color although they were wet, while some of the contaminated particles had a color change in line with what would be expected from observing drying soil. For example, in the photograph below, all four coupons had been treated with $CaCl_2$ and dried for the same amount of time, but obviously they looked very different from one another.<br>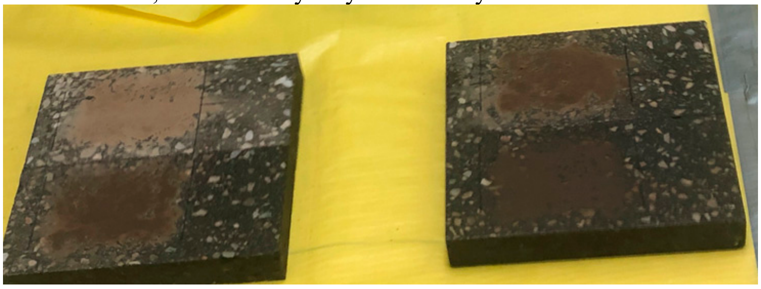<br>This phenomenon made it difficult to be certain that the surfaces were dry since they maintained a wetted appearance; the cause is still not clear. Two possible reasons are air flow patterns in the tent causing different rates of drying or chemical interactions with chemical constituents of the asphalt paver being drawn up into the $CaCl_2$. After 2 to 3 days, the paver surface was touched with a paper towel to confirm dryness and thereafter the experiments were performed.<br><br>**Concrete:** $CaCl_2$ was applied as described above, and it dried to the touch overnight with no other notable observations (see photograph at left below).<br>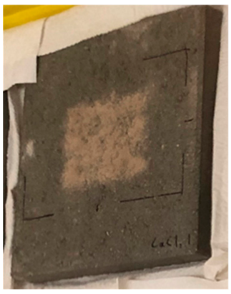 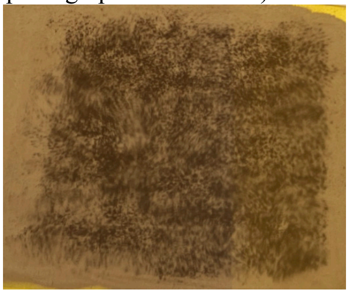<br>**Soil:** $CaCl_2$ was applied as described above, and it dried to the touch overnight with no other notable observations (see photograph at right above). During the walking path experiment, the $CaCl_2$ formed a crust on the soil after drying. This crust tended to bind to the boot during walking and did not release until the walker reached the nonsoil surface.
Health and safety concerns	$CaCl_2$ is stable under normal conditions of use and storage. While use directly in the environment should be evaluated carefully, according to the Safety Data Sheet (SDS), the product is minimally toxic and, upon disposal, is not a hazardous waste according to the Resource Conservation and Recovery Act.
Cost	\$1.45/kg of $CaCl_2$ pellets, which was equivalent to \$1.50/m^2 of testing surface.

Table S2. Phos-Chek MVP-Fx Operational Information**.**

Parameters	Operational Information
Application	**MVP-Fx** was prepared as an aqueous solution (with a viscosity and red color similar to tomato juice – slightly more viscous than water) and applied using a 0.5-gallon handheld garden sprayer as described above. The 2-gallon sprayer used for the other two stabilization technologies would clog when using MVP-Fx. No clog was observed using a 0.5-gallon handheld garden sprayer for MVP-Fx due to different size of orifices for the nozzles. The aqueous solution was applied gently not to disturb the particles on the surface until the particles were fully covered with solution. Likely because of the viscosity of the solution, pooling on the surface occurred during application. The stabilization technologies were allowed to dry until dry to the touch (2-3 days) before experiments were performed.
Surface interactions	**Asphalt:** MVP-Fx was applied as described above, and it dried to the touch within 2-3 days. As it dried, MVP-Fx seemed to remain pooled on the surface of the asphalt and particles, creating a thicker red coating (in comparison to concrete and soil) on the surface when dry (see photograph at left below). 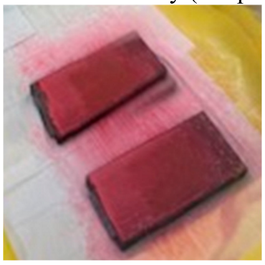 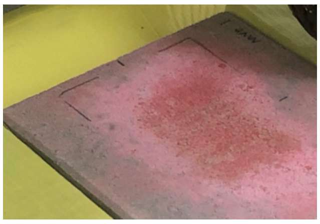 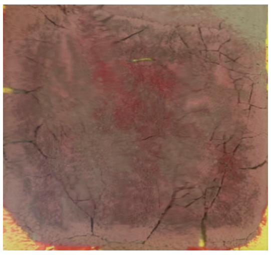 **Concrete:** MVP-Fx was applied as described above, and it dried to the touch within 2-3 days. As it dried, MVP-Fx seemed to penetrate exposed areas of concrete surface and sorb to surface particles resulting in a light red color on the surface (see center photograph above). **Soil:** MVP-Fx was applied as described above, and it dried to the touch within 2-3 days. As it dried, MVP-Fx seemed to penetrate the soil and contaminated particles resulting in a very light red color on the soil surface (see photograph at right above).
Health and safety concerns	Phos-chek MVP-Fx is a fire retardant used for wildland fire control. It is stable under normal conditions. The SDS for Phos-Chek MVP-Fx shows that it may cause long-term adverse effects in the aquatic environment, in general, the product seems to be minimally toxic. There is minimal disposal guidance in the SDS.
Cost	\$6.80/kg of MVP-Fx, which was equivalent to \$5/m^2 of testing surface.

Table S3. $Soil_2O$ Operational Information.

Parameters	Operational Information
Application	$Soil_2O$ was prepared as a dilute aqueous solution (with a viscosity similar to water) and applied using a 2-gallon garden sprayer as described above. The aqueous solution was applied gently not to disturb the particles on the surface until the particles were fully wetted but before pooling on the surface occurred. The stabilization technologies were allowed to dry until dry to the touch (at least overnight) before experiments were performed.
Surface interactions	$Soil_2O$ was applied to asphalt, concrete, and soil and it dried to the touch overnight with no other notable observations (see photographs below). 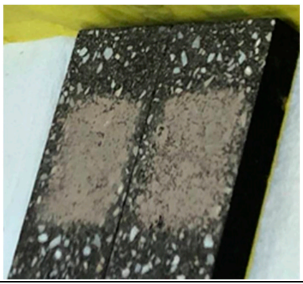 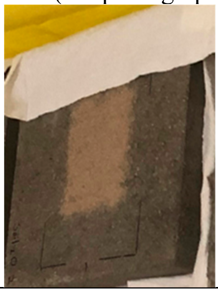 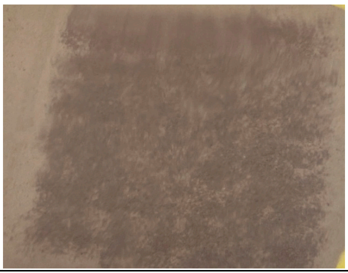
Health and safety concerns	$Soil_2O^{TM}$ is a dust control agent. It is stable under normal conditions. According to the SDS, it seems to be a minimally toxic product that has been shown to generate no negative or toxic effects on the environment when released in dilution for terrestrial and aquatic ecosystems. It is a nonhazardous waste material suitable for approved solid waste landfills, as it is has demonstrated to be immobile in landfills and soil systems.
Cost	\$3.47/kg of $Soil_2O$, which was equivalent to \$0.28/m^2 of testing surface.
